# Supplementary material for: Ion selectivity and rotor coupling of the Vibrio flagellar sodium-driven stator unit
Source: Nat Commun. 2023 Jul 27;14:4411. doi: 10.1038/s41467-023-39899-z (PMC10374538; doi:10.1038/s41467-023-39899-z)
Supplement: Supplementary file 1 — Supplementary information [file 41467_2023_39899_MOESM1_ESM.pdf]

Supplementary Information

Ion selectivity and rotor coupling of the *Vibrio* flagellar sodium-driven stator unit

Haidai Hu[1], Philipp F. Popp[2], Mònica Santiveri[1], Aritz Roa-Eguiara[1], Yumeng Yan[1], Freddie J. O. Martin[1], Zheyi Liu[3,4], Navish Wadhwa[5,6], Yong Wang[3,4], Marc Erhardt[2,7], Nicholas M. I. Taylor[1]*

[1]Structural Biology of Molecular Machines Group, Protein Structure & Function Program, Novo Nordisk Foundation Center for Protein Research, Faculty of Health and Medical Sciences, University of Copenhagen, Blegdamsvej 3B, 2200 Copenhagen, Denmark.

[2]Institute for Biology/Molecular Microbiology, Humboldt-Universität zu Berlin, Philippstr. 13, 10115 Berlin, Germany.

[3]College of Life Sciences, Zhejiang University, Hangzhou 310027, China.

[4]The Provincial International Science and Technology Cooperation Base on Engineering Biology, International Campus of Zhejiang University, Haining, 314400, China.

[5]Department of Physics, Arizona State University, Tempe, AZ, 85287, USA.

[6]Biodesign Center for Mechanisms of Evolution, Arizona State University, Tempe, AZ, 85287, USA.

[7]Max Planck Unit for the Science of Pathogens, Berlin, Germany.

*Correspondence: nicholas.taylor@cpr.ku.dk

This PDF file includes:

Supplementary Figures 1-16

Supplementary Tables 1-3

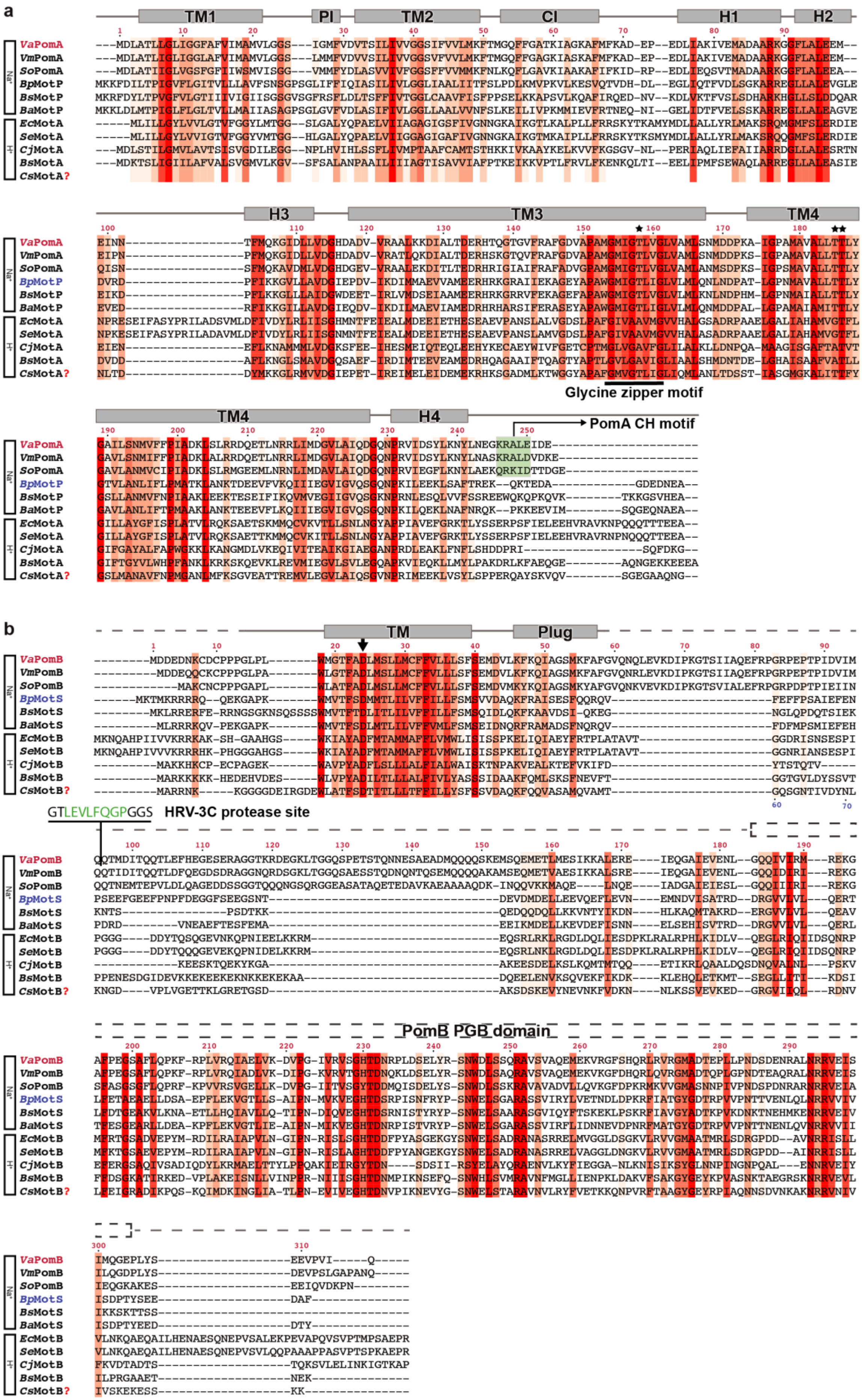
a
TM1
PI
TM2
CI
H1
H2
H3
TM3
TM4
H4
PomA CH motif
Glycine zipper motif
VaPomA
VmPomA
SoPomA
BpMotP
BsMotP
BaMotP
EcMotA
SeMotA
CjMotA
BsMotA
CsMotA?
Na+
H+
b
TM
Plug
GTLEVLFQGPGGS
HRV-3C protease site
PomB PGB domain
VaPomB
VmPomB
SoPomB
BpMotS
BsMotS
BaMotS
EcMotB
SeMotB
CjMotB
BsMotB
CsMotB?

Supplementary Fig. 1. Protein sequence alignment of *Va*PomA and *Va*PomB homologs from different bacterial species.

a-**b**, Multiple-sequence alignment of PomA (**a**) and PomB (**b**). The proteins are grouped into two families: sodium- and proton- driven stator units. In the case of *Cs*MotAB, whose cryo-EM structure is available, the ion type is ambiguous, and therefore it is labeled with a question mark. *Va*PomAB residue numbers (in red) are given above the sequences. Helices are indicated by solid boxes. Residues that are identical or partially conserved are highlighted in red and orange, respectively. Residues that are critical for sodium ion selectivity in PomAB (T158, T185 and T186) are marked with a star. Dashed line above the PomB sequence indicates that the structure was not resolved in the PomAB complex cryo-EM map. PomB PGB domain and HRV-3C protease site are also indicated above the sequence alignment. PomA C-terminal helical motif is highlighted by a semi-transparent green box. Sequences aligned: *Vibrio alginolyticus Va*PomAB; *Vibrio mimicus Vm*PomAB; *Shewanella oneidensis So*PomA *and So*PomB; *Bacillus pseudofirmus Bp*MotPS; *Bacillus subtilis Bs*MotPS, *Bs*MotAB; *Bacillus alcalophilus Ba*MotPS; *Escherichia coli Ec*MotAB; *Salmonella enterica Se*MotAB; *Campylobacter jejuni Cj*MotAB; *Clostridium sporogenes Cs*MotAB.

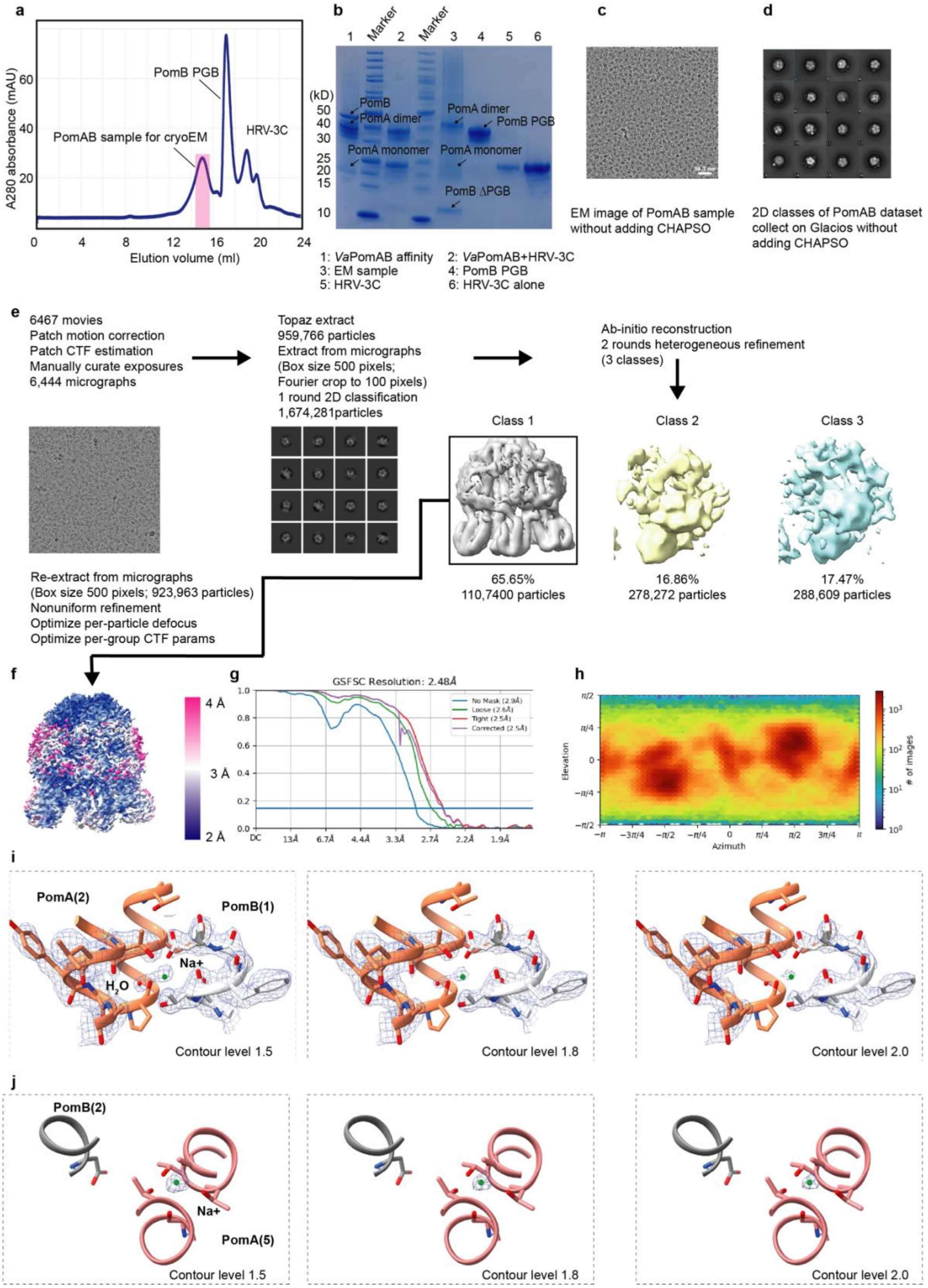
a
A280 absorbance (mAU)
Elution volume (ml)
PomB PGB
PomAB sample for cryoEM
HRV-3C
b
Marker
(kD)
PomB
PomA dimer
PomA monomer
PomB PGB
PomB ΔPGB
1: VaPomAB affinity
2: VaPomAB+HRV-3C
3: EM sample
4: PomB PGB
5: HRV-3C
6: HRV-3C alone
c
EM image of PomAB sample without adding CHAPSO
d
2D classes of PomAB dataset collect on Glacios without adding CHAPSO
e
6467 movies
Patch motion correction
Patch CTF estimation
Manually curate exposures
6,444 micrographs
Topaz extract
959,766 particles
Extract from micrographs
(Box size 500 pixels;
Fourier crop to 100 pixels)
1 round 2D classification
1,674,281particles
Ab-initio reconstruction
2 rounds heterogeneous refinement
(3 classes)
Class 1
Class 2
Class 3
65.65%
110,7400 particles
16.86%
278,272 particles
17.47%
288,609 particles
Re-extract from micrographs
(Box size 500 pixels; 923,963 particles)
Nonuniform refinement
Optimize per-particle defocus
Optimize per-group CTF params
f
4 Å
3 Å
2 Å
g
GSFSC Resolution: 2.48Å
No Mask (2.9Å)
Loose (2.6Å)
Tight (2.5Å)
Corrected (2.5Å)
h
Elevation
Azimuth
# of images
i
PomA(2)
PomB(1)
Na+
H2O
Contour level 1.5
Contour level 1.8
Contour level 2.0
j
PomB(2)
Na+
PomA(5)
Contour level 1.5
Contour level 1.8
Contour level 2.0

Supplementary Fig. 2. Cryo-EM of *Va*PomAB in LMNG detergent.

a, A representative SEC profile of LMNG detergent purified *Va*PomAB complex. The fraction used for preparing cryo-EM grids is indicated with a pink rectangular bar. **b**, SDS gel from **a** is shown. Gel is a representative that consistently yields similar results at least three times. **c-d**, EM dataset of *Va*PomAB without adding CHAPSO. The image is one representative among approximately 1000 images. **e-f**, Flowchart of the data processing of *Va*PomAB in LMNG in cryoSPARC that results in the final cryo-EM structure of *Va*PomAB at around 2.5 Å resolution after non-uniform refinement, with **f** shows cryo-EM density map of *Va*PomAB in LMNG detergent colored by local resolution (in Å) estimated in cryoSPARC. **g**, Gold standard (0.143) Fourier shell correlation (GSFSC) curves for *Va*PomAB in LMNG. **h**, Particle directional distribution of *Va*PomAB in LMNG. **i-j**, Representative model segments fitted into EM density, focusing on the Na^+ binding sites, with different contour level.

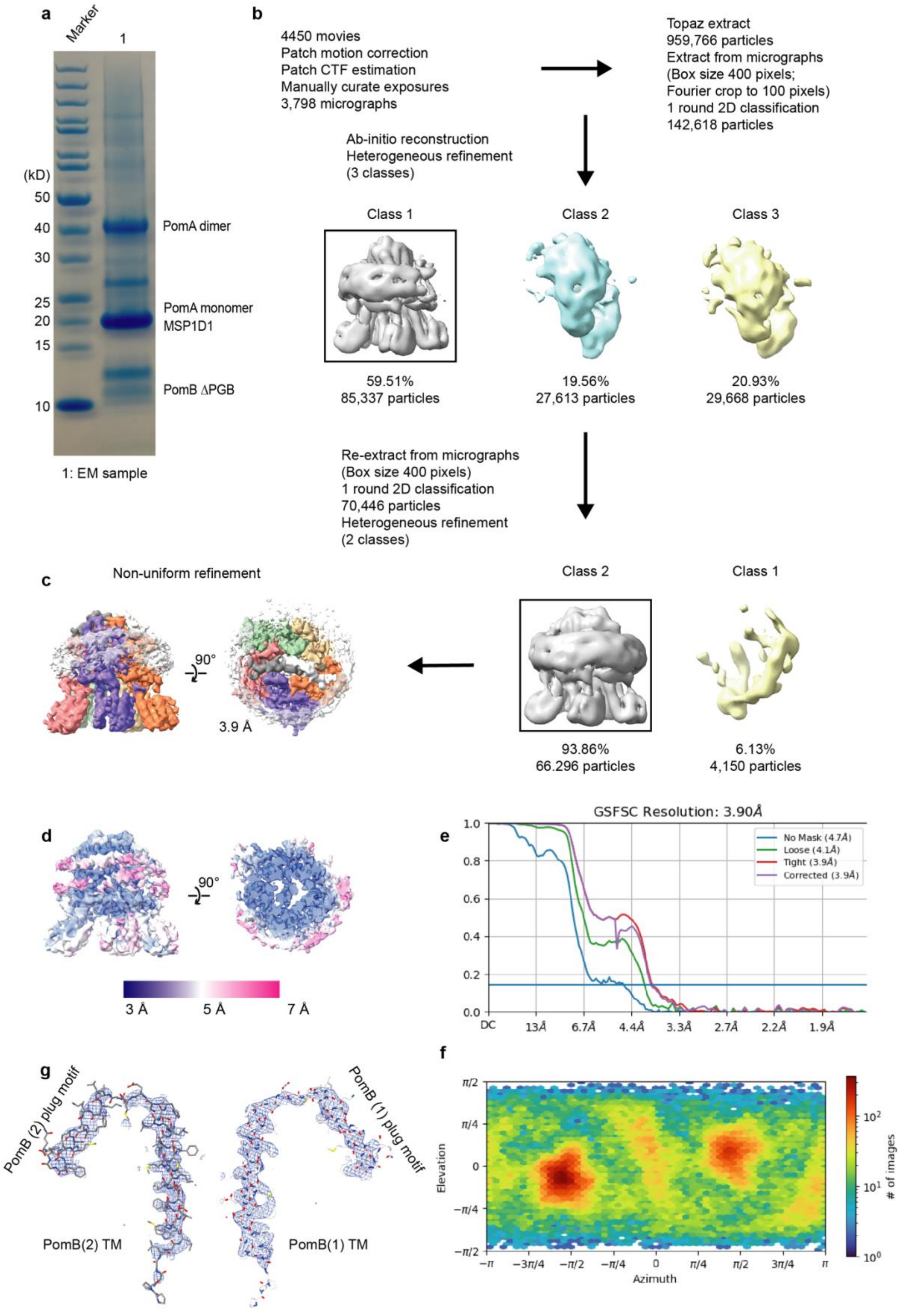
a
Marker
1
(kD)
50
40
30
25
20
15
10
PomA dimer
PomA monomer
MSP1D1
PomB ΔPGB
1: EM sample
b
4450 movies
Patch motion correction
Patch CTF estimation
Manually curate exposures
3,798 micrographs
Topaz extract
959,766 particles
Extract from micrographs
(Box size 400 pixels;
Fourier crop to 100 pixels)
1 round 2D classification
142,618 particles
Ab-initio reconstruction
Heterogeneous refinement
(3 classes)
Class 1
Class 2
Class 3
59.51%
85,337 particles
19.56%
27,613 particles
20.93%
29,668 particles
Re-extract from micrographs
(Box size 400 pixels)
1 round 2D classification
70,446 particles
Heterogeneous refinement
(2 classes)
c
Non-uniform refinement
90°
3.9 Å
Class 2
Class 1
93.86%
66.296 particles
6.13%
4,150 particles
d
90°
3 Å
5 Å
7 Å
e
GSFSC Resolution: 3.90Å
No Mask (4.7Å)
Loose (4.1Å)
Tight (3.9Å)
Corrected (3.9Å)
DC
13Å
6.7Å
4.4Å
3.3Å
2.7Å
2.2Å
1.9Å
f
Elevation
Azimuth
# of images
g
PomB (2) plug motif
PomB (1) plug motif
PomB(2) TM
PomB(1) TM

Supplementary Fig. 3. Cryo-EM of *Va*PomAB in MSP1D1 lipid nanodisc.

a, SDS gel analysis of purified *Va*PomAB in MSP1D1 lipid nanodisc. Gel is a representative that consistently yields similar results at least three times. **b**, Flowchart of the data processing of *Va*PomAB in MSP1D1 lipid nanodisc in cryoSPARC that results in the final cryo-EM structure. **c**, The final cryo-EM map of *Va*PomAB in MSP1D1 lipid nanodisc at around 3.9 Å resolution. **d**, Cryo-EM density map of *Va*PomAB in MSP1D1 lipid nanodisc colored by local resolution (in Å) estimated in cryoSPARC. **e**, Gold standard (0.143) Fourier shell correlation (GSFSC) curves for *Va*PomAB in MSP1D1 lipid nanodisc. **f**, Particle directional distribution of *Va*PomAB in MSP1D1nanodisc. **g**, Representative model segments fitted into EM density.

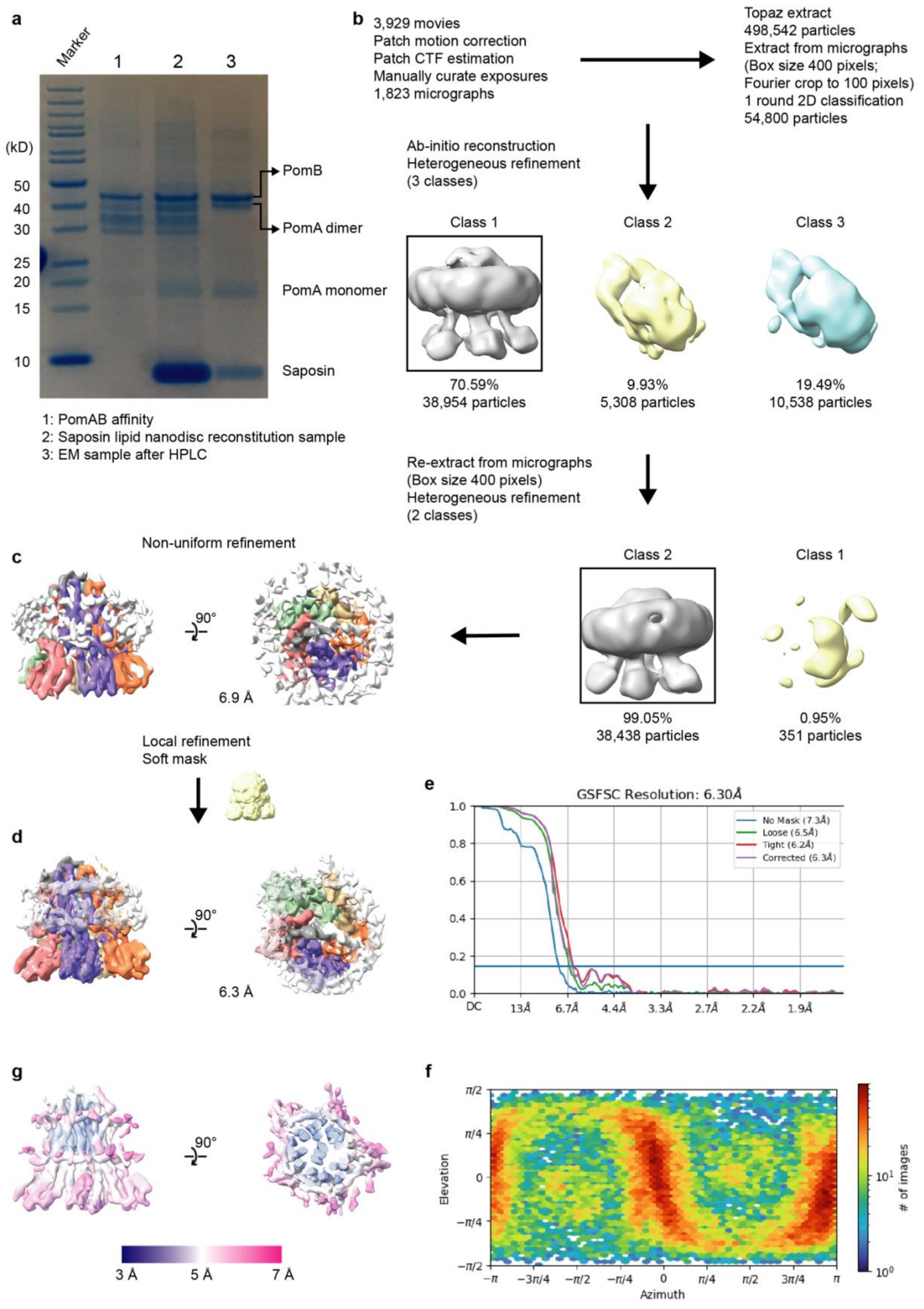
a
Marker
1
2
3
(kD)
50
40
30
25
20
15
10
PomB
PomA dimer
PomA monomer
Saposin
1: PomAB affinity
2: Saposin lipid nanodisc reconstitution sample
3: EM sample after HPLC
b
3,929 movies
Patch motion correction
Patch CTF estimation
Manually curate exposures
1,823 micrographs
Topaz extract
498,542 particles
Extract from micrographs
(Box size 400 pixels;
Fourier crop to 100 pixels)
1 round 2D classification
54,800 particles
Ab-initio reconstruction
Heterogeneous refinement
(3 classes)
Class 1
Class 2
Class 3
70.59%
38,954 particles
9.93%
5,308 particles
19.49%
10,538 particles
Re-extract from micrographs
(Box size 400 pixels)
Heterogeneous refinement
(2 classes)
Class 2
Class 1
99.05%
38,438 particles
0.95%
351 particles
c
Non-uniform refinement
90°
6.9 Å
Local refinement
Soft mask
d
90°
6.3 Å
e
GSFSC Resolution: 6.30Å
No Mask (7.3Å)
Loose (6.5Å)
Tight (6.2Å)
Corrected (6.3Å)
DC
13Å
6.7Å
4.4Å
3.3Å
2.7Å
2.2Å
1.9Å
g
90°
3 Å
5 Å
7 Å
f
Elevation
Azimuth
# of images

Supplementary Fig. 4. Cryo-EM of full length *Va*PomAB in saposin lipid nanodisc.

a, SDS gel analysis of the purified full length *Va*PomAB in saposin lipid nanodisc. Gel is a representative that consistently yields similar results at least three times. **b**-**c**, Flowchart of the data processing of full length *Va*PomAB in saposin lipid nanodisc in cryoSPARC that results in the final cryo-EM structure. **d**, The final cryo-EM map of *Va*PomAB in saposin lipid nanodisc at around 6.3 Å resolution after local refinement. **e**, Gold standard (0.143) Fourier shell correlation (GSFSC) curves for *Va*PomAB in saposin lipid nanodisc. **f**, Particle directional distribution. **g**, Cryo-EM density map of *Va*PomAB in saposin lipid nanodisc colored by local resolution (in Å) estimated in cryoSPARC.

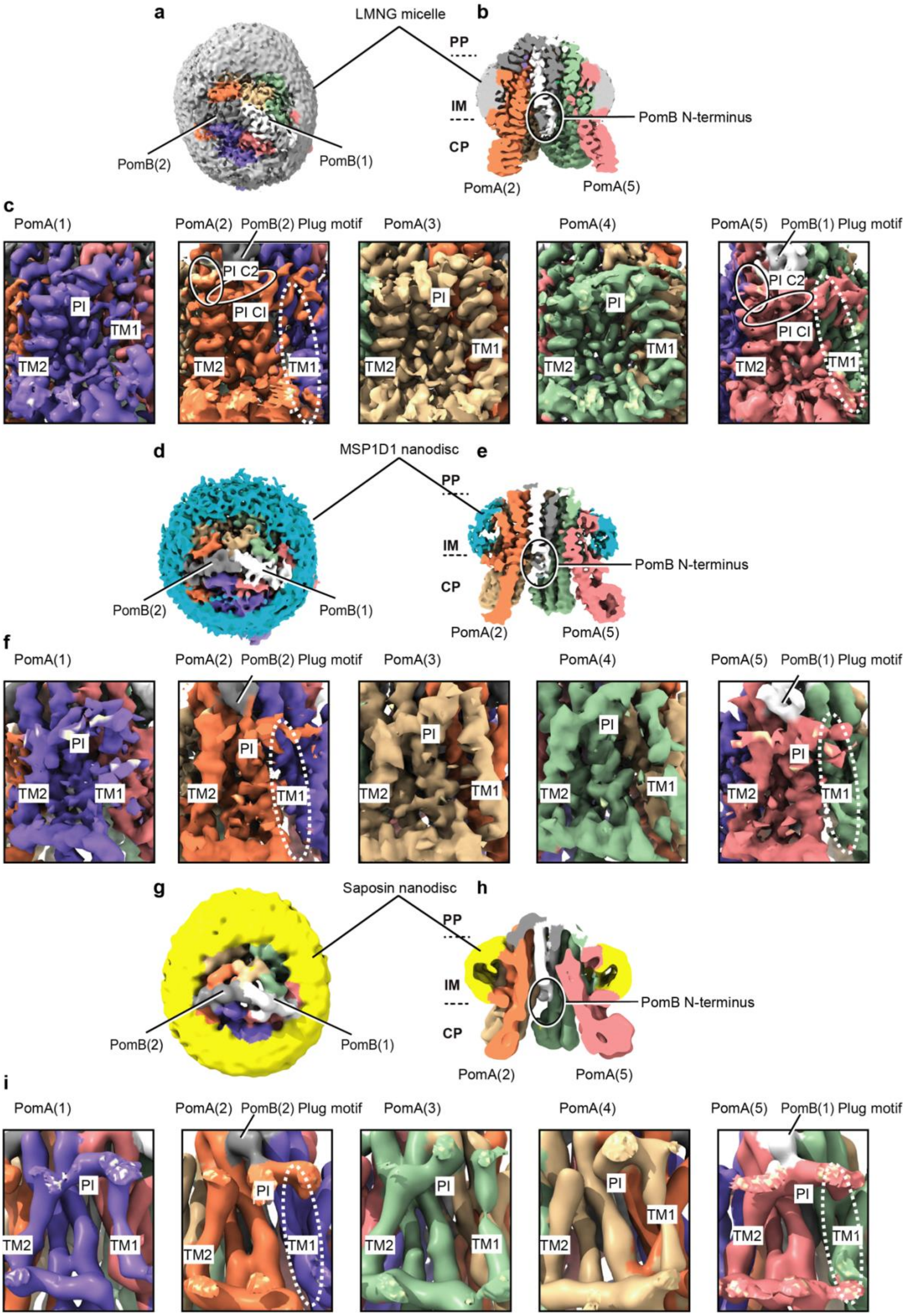

a
b
LMNG micelle
PP
IM
CP
PomB N-terminus
PomB(2)
PomB(1)
PomA(2)
PomA(5)
c
PomA(1)
PomA(2)
PomB(2) Plug motif
PomA(3)
PomA(4)
PomA(5)
PomB(1) Plug motif
PI
PI C2
PI CI
TM1
TM2
d
e
MSP1D1 nanodisc
f
g
h
Saposin nanodisc
i

Supplementary Fig. 5. Dynamics of *Va*PomA PI and TM1 helices.

a-c, Representation of the *Va*PomAB LMNG unsharpened electrostatic potential maps at low threshold showing the conformational dynamic of PI helices that interact with PomB plug motifs, and the flexibility of the corresponding TM1 helices. **d-f**, Representation of the *Va*PomAB MSP1D1 lipid nanodisc unsharpened electrostatic potential maps at low threshold. **g-i**, Representation of the full length *Va*PomAB saposin lipid nanodisc unsharpened electrostatic potential maps at low threshold.

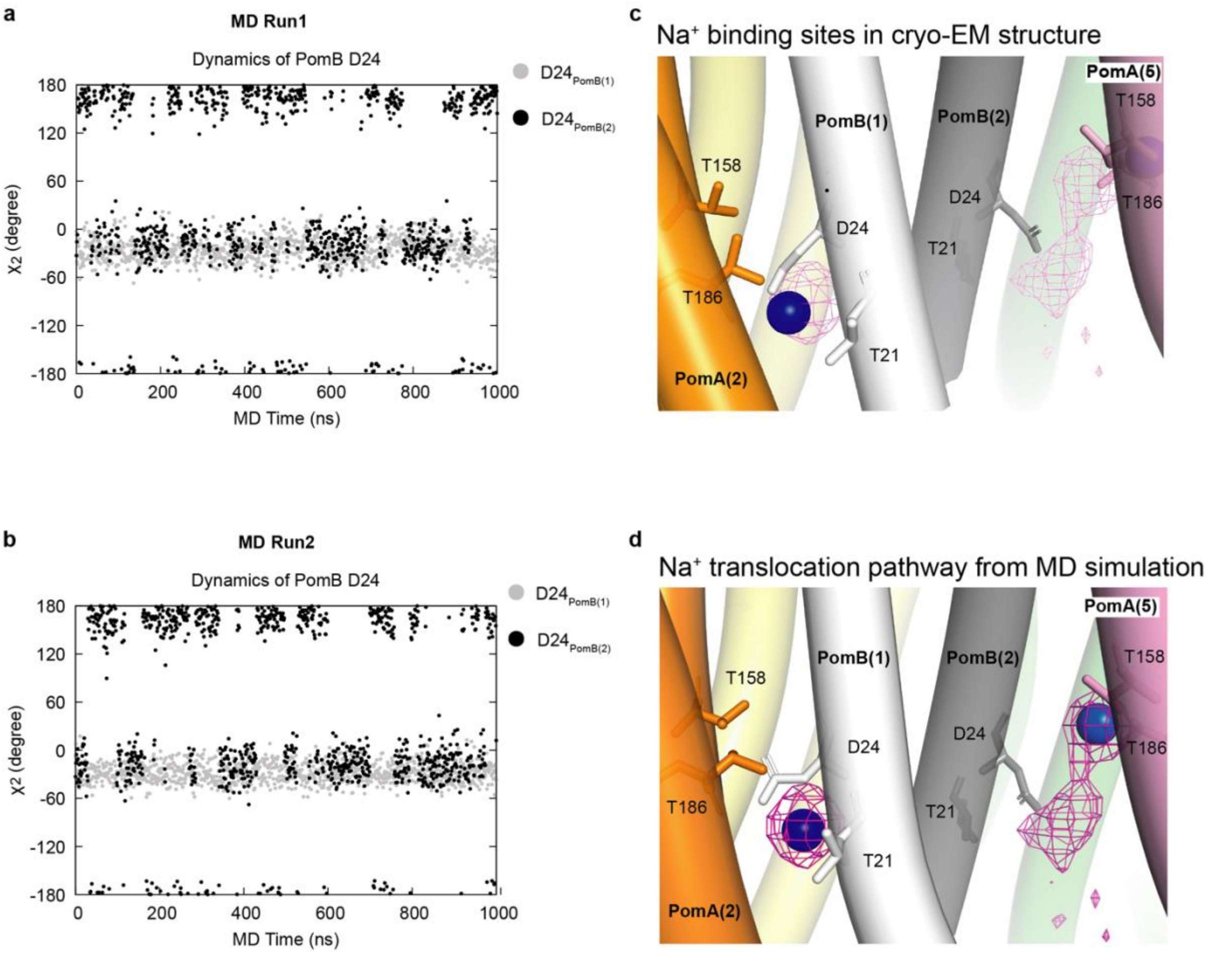

Supplementary Fig. 6. Na^+ translocation pathway and dynamics of PomB D24.

a-**b**, The trajectories of the side chain dynamics of D24 in PomB chain 1 and 2 obtained from two independent MD simulations. The χ2 angle of D24 is the angle between the planes formed by Cα-Cβ-Cγ and Cβ-Cγ-Oδ atoms. **c**, The cryo-EM Na^+ binding sites. The modelled Na^+ ions are shown by blue spheres. **d**, The Na^+ binding sites captured in MD simulations. The average density of Na^+ ions is represented by red mesh in **c** and **d**.

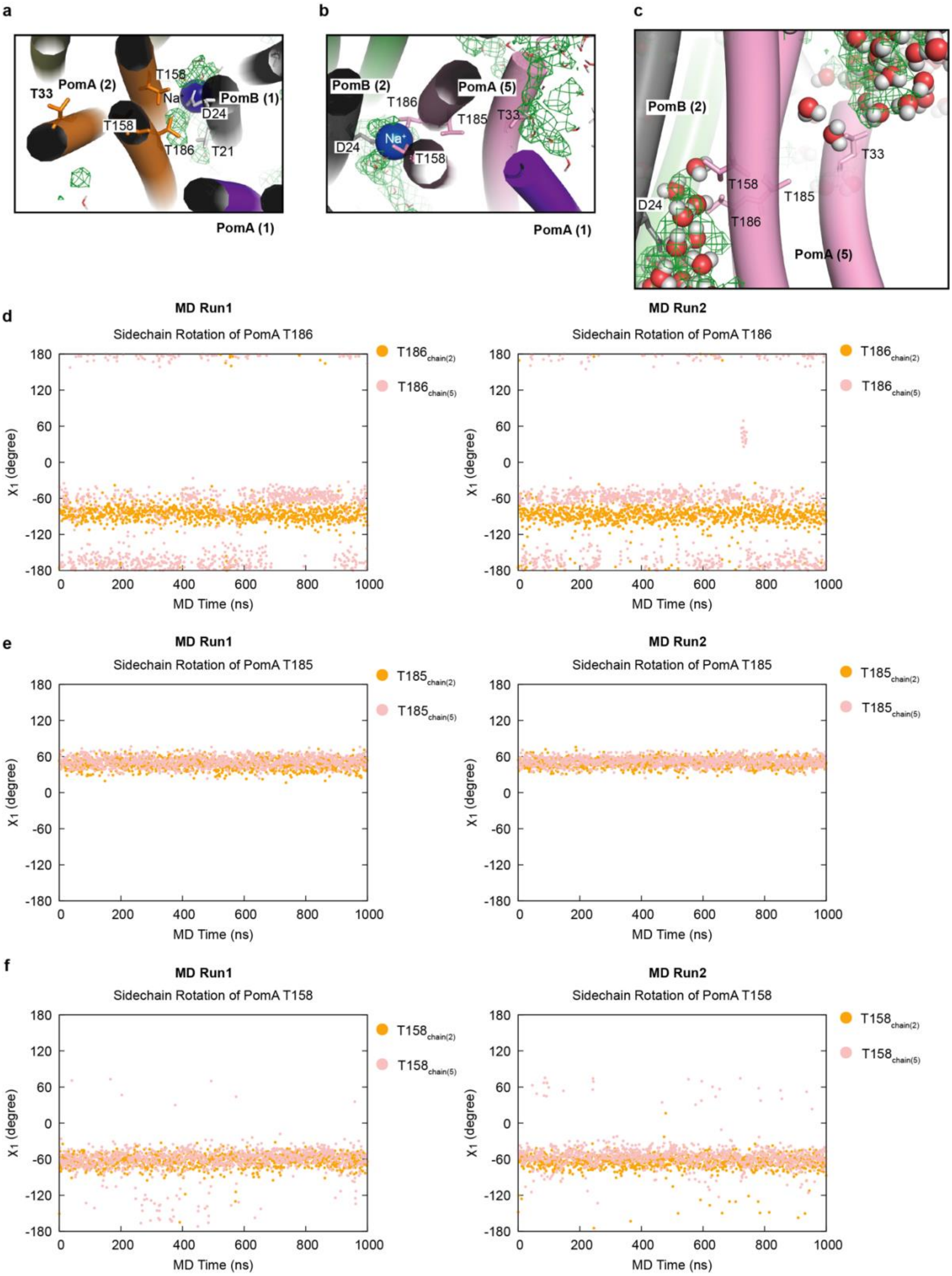
a
T33
PomA (2)
T158
Na
PomB (1)
D24
T158
T186
T21
PomA (1)
b
PomB (2)
PomA (5)
T186
T185
T33
D24
Na+
T158
PomA (1)
c
PomB (2)
T33
T158
T185
D24
T186
PomA (5)
d
MD Run1
Sidechain Rotation of PomA T186
MD Run2
Sidechain Rotation of PomA T186
T186chain(2)
T186chain(5)
X1 (degree)
MD Time (ns)
e
MD Run1
Sidechain Rotation of PomA T185
MD Run2
Sidechain Rotation of PomA T185
T185chain(2)
T185chain(5)
X1 (degree)
MD Time (ns)
f
MD Run1
Sidechain Rotation of PomA T158
MD Run2
Sidechain Rotation of PomA T158
T158chain(2)
T158chain(5)
X1 (degree)
MD Time (ns)

Supplementary Fig. 7. Hydration of T33 and the Na^+ translocation pathway and side chain dynamics of T158, T185 and T186 obtained from explicit solvent MD simulations.

a-**b**, The hydration and Na^+ binding in the engaged and disengaged state, respectively. The average density of water molecules is represented by mesh in green. **c**, A snapshot from the MD simulations to show the hydration of T33 in PomA chain 5. **d**-**f**, The MD trajectories of the side chain dynamics of T186, T185 and T158 in PomA chain 2 and 5. The $\chi 1$ angle of T158, T185 and T186 is the angle between the planes formed by N-Cα-Cβ and Cα-Cβ-Oγ atoms.

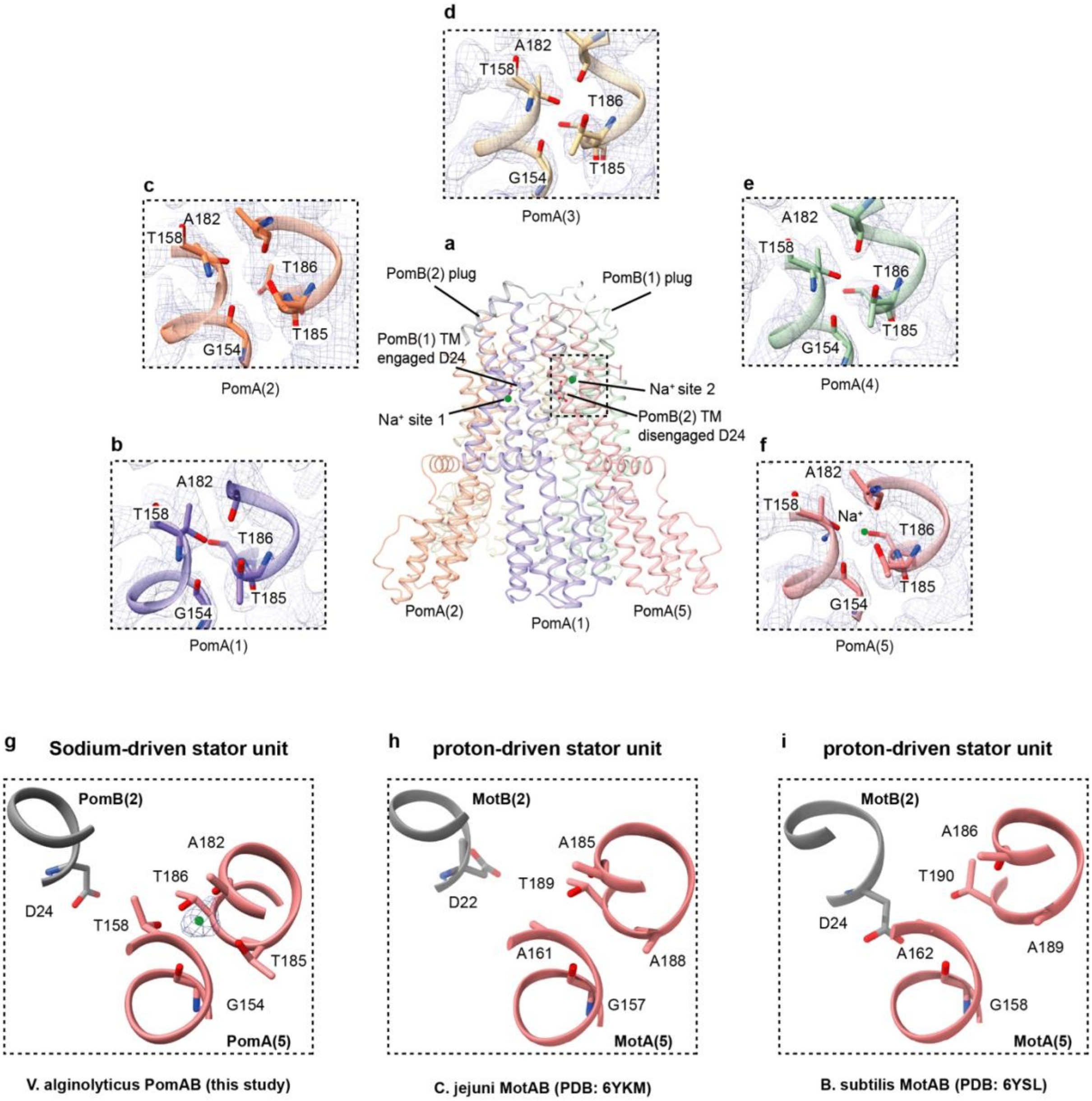

Supplementary Fig. 8. densities of ion selectivity cavities.

a, View from the plane of the membrane, showing the position of ion selectivity cavity within the complex. **b-f**, ion selectivity cavities from PomA chains 1 to 5. EM densities are overlaid on the corresponding local regions. **g-i**, Structure comparations of the Na^+ binding site 2 in *V. alginolyticus* PomAB, with the corresponding sites in *C. jejuni* MotAB and *B. subtilis* MotAB.

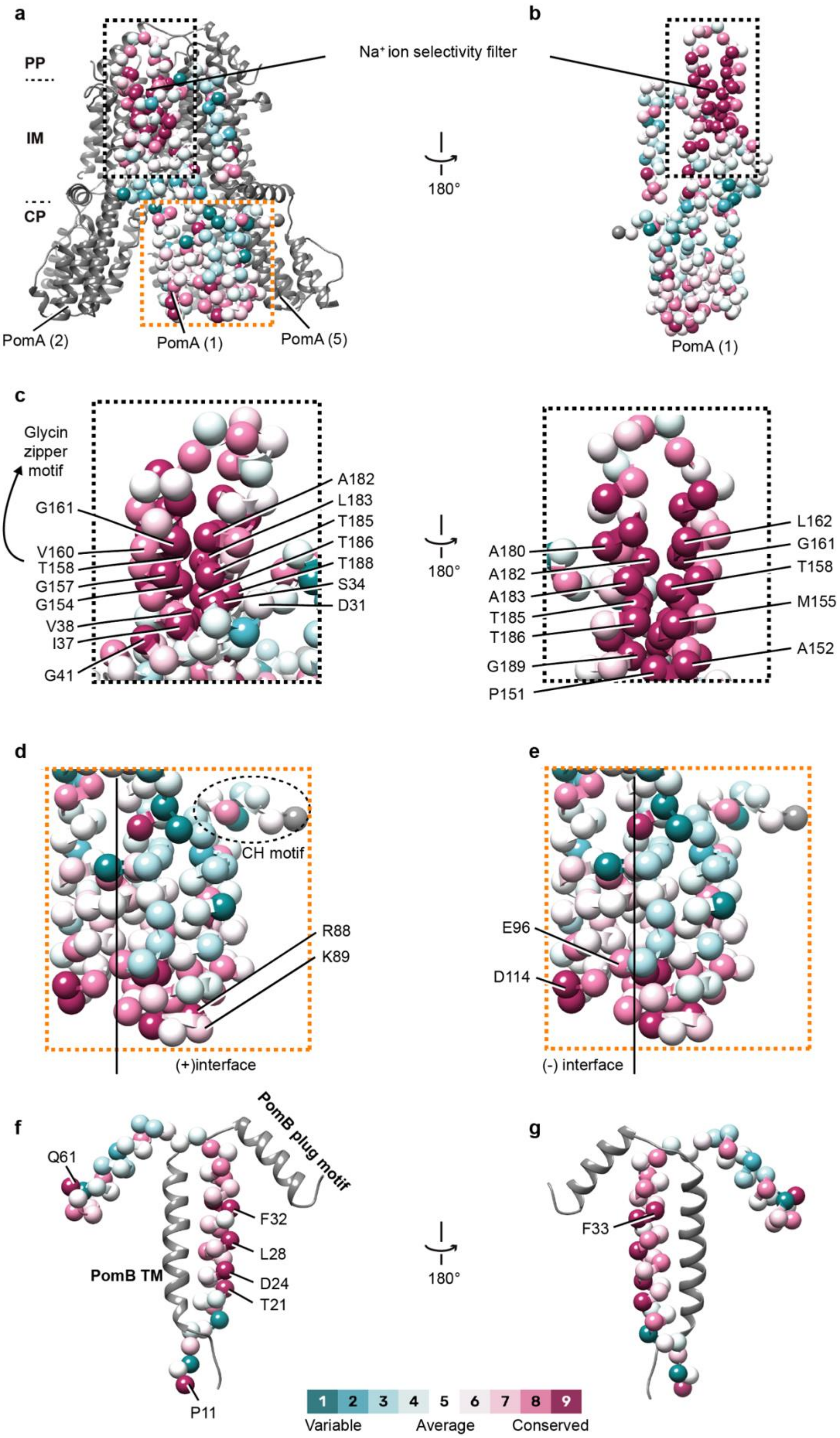
a
PP
IM
CP
PomA (2)
PomA (1)
PomA (5)
Na⁺ ion selectivity filter
180°
b
PomA (1)
c
Glycin
zipper
motif
G161
V160
T158
G157
G154
V38
I37
G41
A182
L183
T185
T186
T188
S34
D31
180°
A180
A182
A183
T185
T186
G189
P151
L162
G161
T158
M155
A152
d
CH motif
R88
K89
(+)interface
e
E96
D114
(-) interface
f
Q61
PomB plug motif
F32
L28
D24
T21
PomB TM
P11
180°
g
F33
1
2
3
4
5
6
7
8
9
Variable
Average
Conserved

Supplementary Fig. 9. Conservation (calculated with ConSurf) analysis of *Va*PomA and *Va*PomB.

a-**b**, Conservation (calculated with ConSurf) of the surface residues of *Va*PomA from external and internal sides; Cα atom representation (shown as spheres) of the model colored by conservation. **c**, Conservation of the residues of the Na^+ ion selectivity filter and permeation pathway from the periplasmic side, both external and internal views are shown. **d**, Conservation of the residues of PomA cytoplasmic domain, highlighting the locations of the positively charged residues from the principal face involved in FliG torque helix binding. **e**, Same as in **d**, but highlighting negatively charged residues from the complementary face. **f**, Conservation of the surface residues of *Va*PomB, highlighting the strictly conserved residues. **g**, Same as in **f**, but rotated 180 degrees.

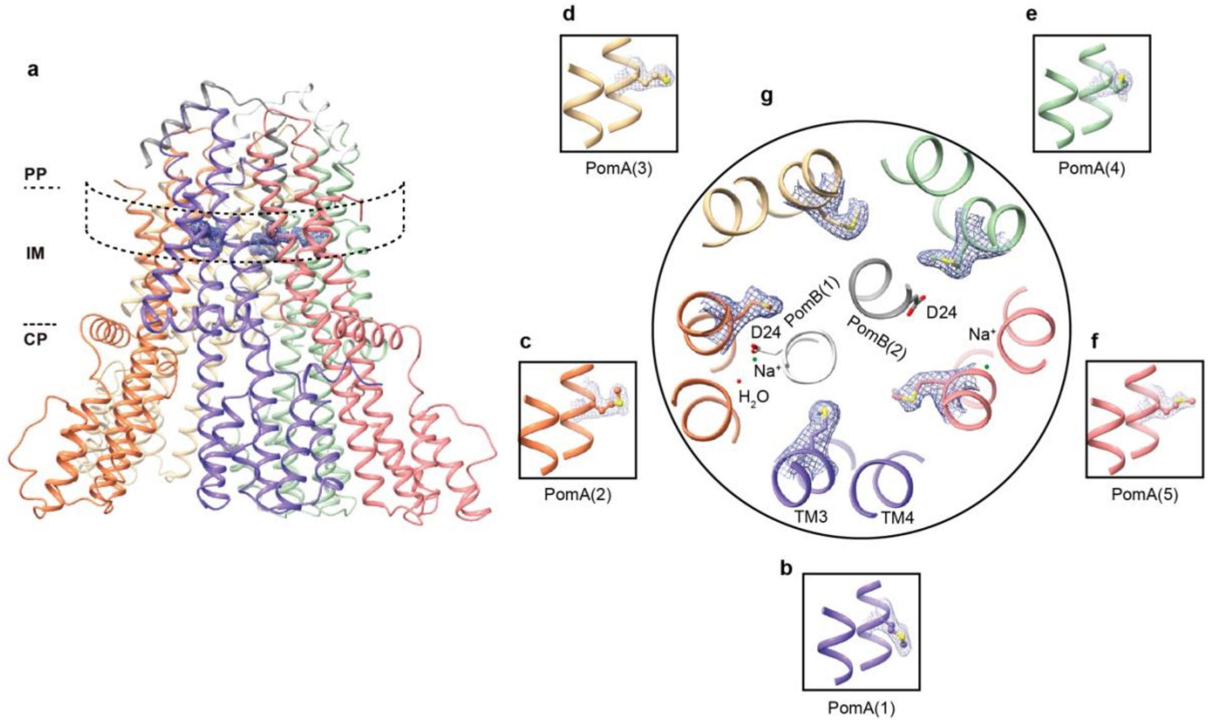

Supplementary Fig. 10. Conformational isomers of *Va*PomAB M155.

a, View from the plane of the membrane, showing the position of PomA M155 within the complex. **b**-**f**, M155 isomers from PomA chains 1 to 5. EM densities are overlaid on the side chains of M155. **g**, Conformational isomers of M155 viewed from the top of the membrane.

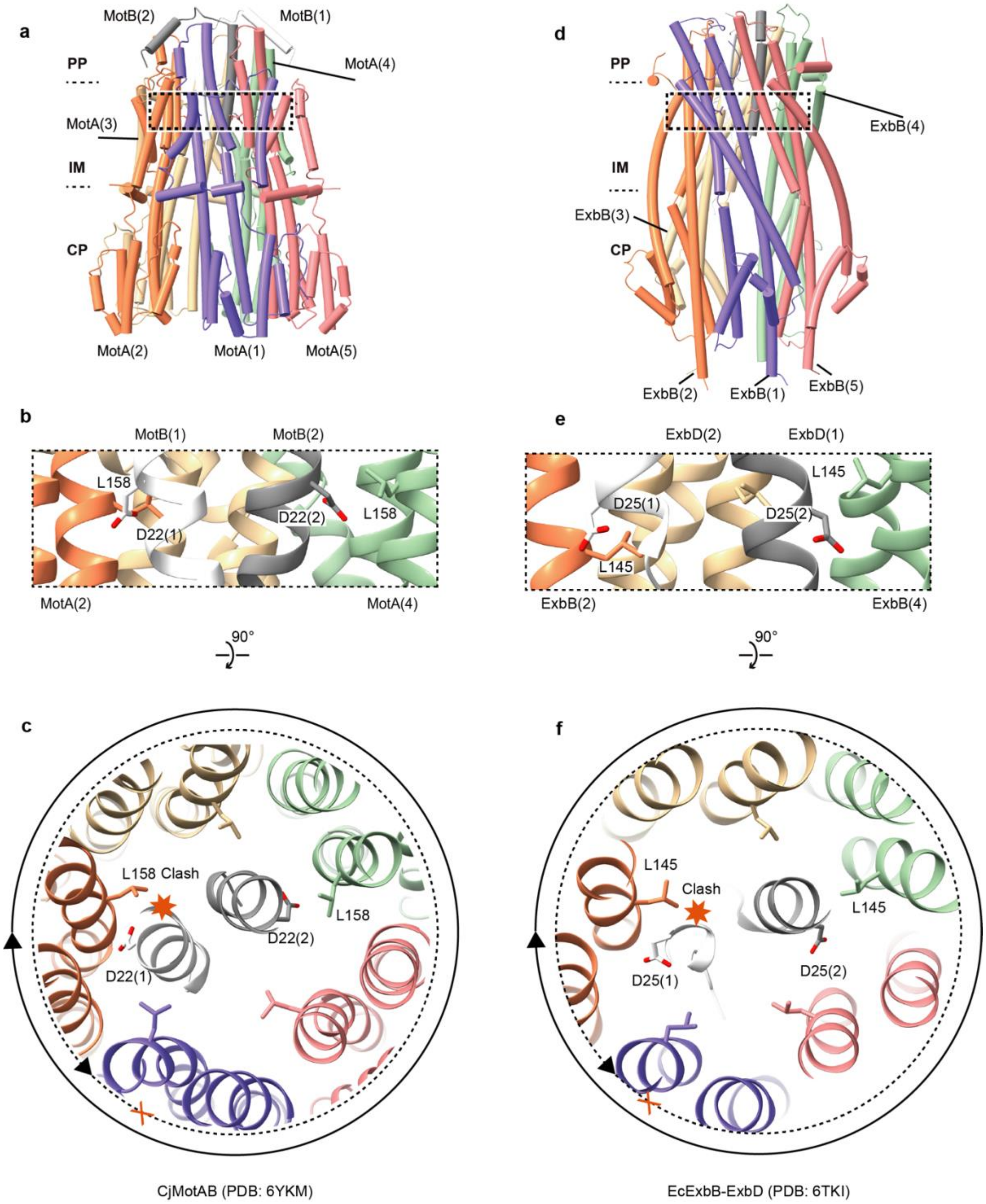
a
MotB(2)
MotB(1)
PP
MotA(4)
MotA(3)
IM
CP
MotA(2)
MotA(1)
MotA(5)
b
MotB(1)
MotB(2)
L158
D22(1)
D22(2)
L158
MotA(2)
MotA(4)
90°
c
L158 Clash
L158
D22(1)
D22(2)
CjMotAB (PDB: 6YKM)
d
PP
ExbB(4)
IM
ExbB(3)
CP
ExbB(2)
ExbB(1)
ExbB(5)
e
ExbD(2)
ExbD(1)
L145
D25(1)
D25(2)
L145
ExbB(2)
ExbB(4)
90°
f
L145
Clash
L145
D25(1)
D25(2)
EcExbB-ExbD (PDB: 6TKI)

Supplementary Fig. 11. 5:2 rotary motor directional rotation 'reinforcement' point.

a, Proton-driven flagellar stator unit *Cj*MotAB (PDB: 6YKM). **b**, Conformational isomers of L158 near MotB engaged D24 and disengaged D24. **c**, Conformational isomers of L158 viewed from the top of the membrane. Solid circle indicates the rotational direction of MotA around MotB. The potential clash that would occur if PomA rotated CCW around PomB is indicated with a red heptagon. **d**, Proton-driven Ton ExbB-ExbD complex (PDB: 6TKI). **e**, Conformational isomers of L145 near ExbD engaged D25 and disengaged D25. **f**, Conformational isomers of ExbB L145 viewed from the top of the membrane. Solid circle indicates the rotational direction of ExbB around ExbD. The potential clash that would occur if ExbB rotated CCW around ExbD is indicated with a red heptagon.

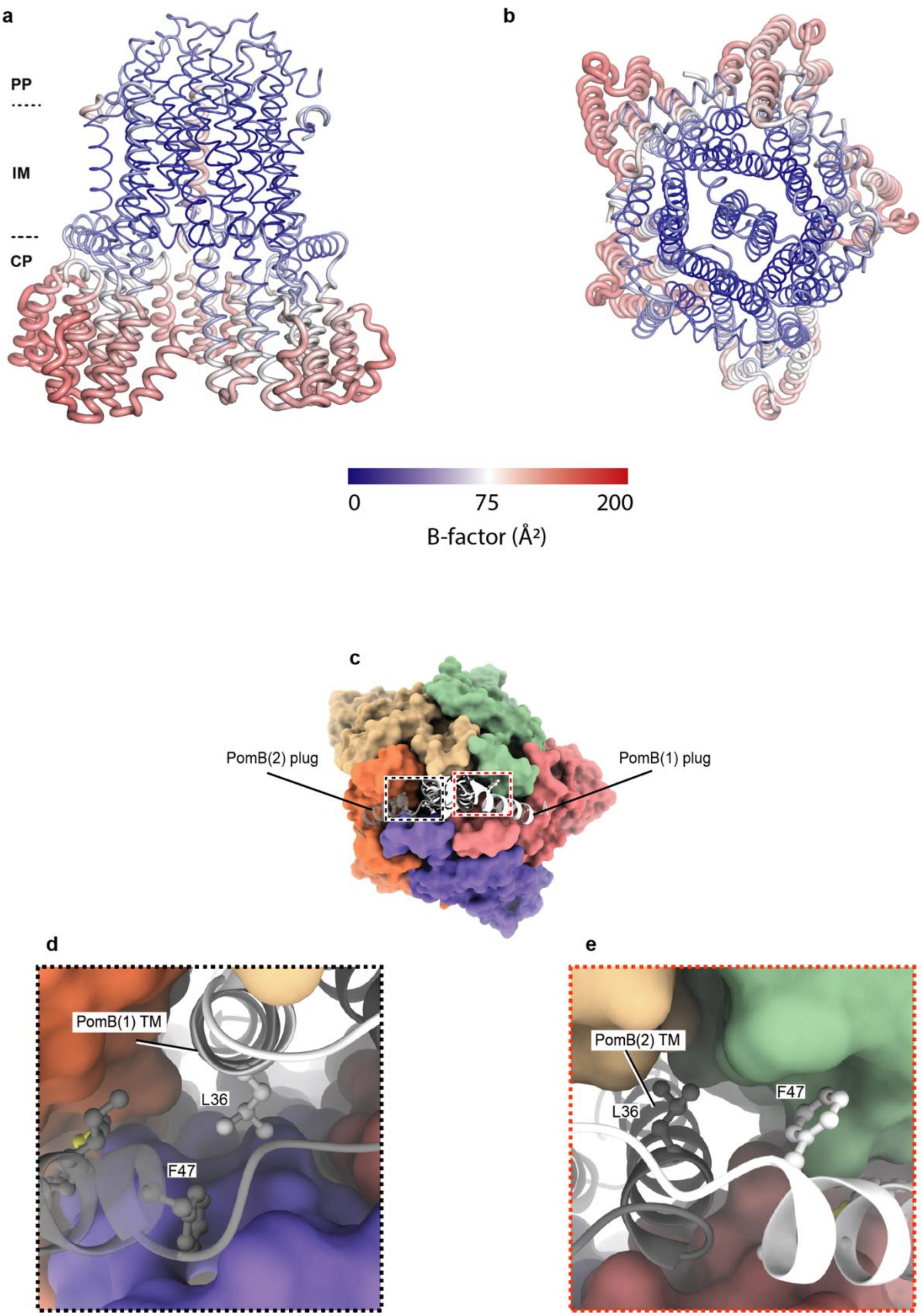
a
PP
IM
CP
b
0
75
200
B-factor (Å²)
c
PomB(2) plug
PomB(1) plug
d
PomB(1) TM
L36
F47
e
PomB(2) TM
L36
F47

Supplementary Fig. 12. *Va*PomAB model B-factor distribution.

Top (**a**) and side views (**b**) of the PomAB model (LMNG dataset) colored by B-factor distribution (atomic displacement factor). **c-e,** L36 from PomB chain 1 (**d**) and chain 2 (**e**) interaction environments, showing that PomB chain 1 L36 interacts PomB chain 2 F47 (**d**).

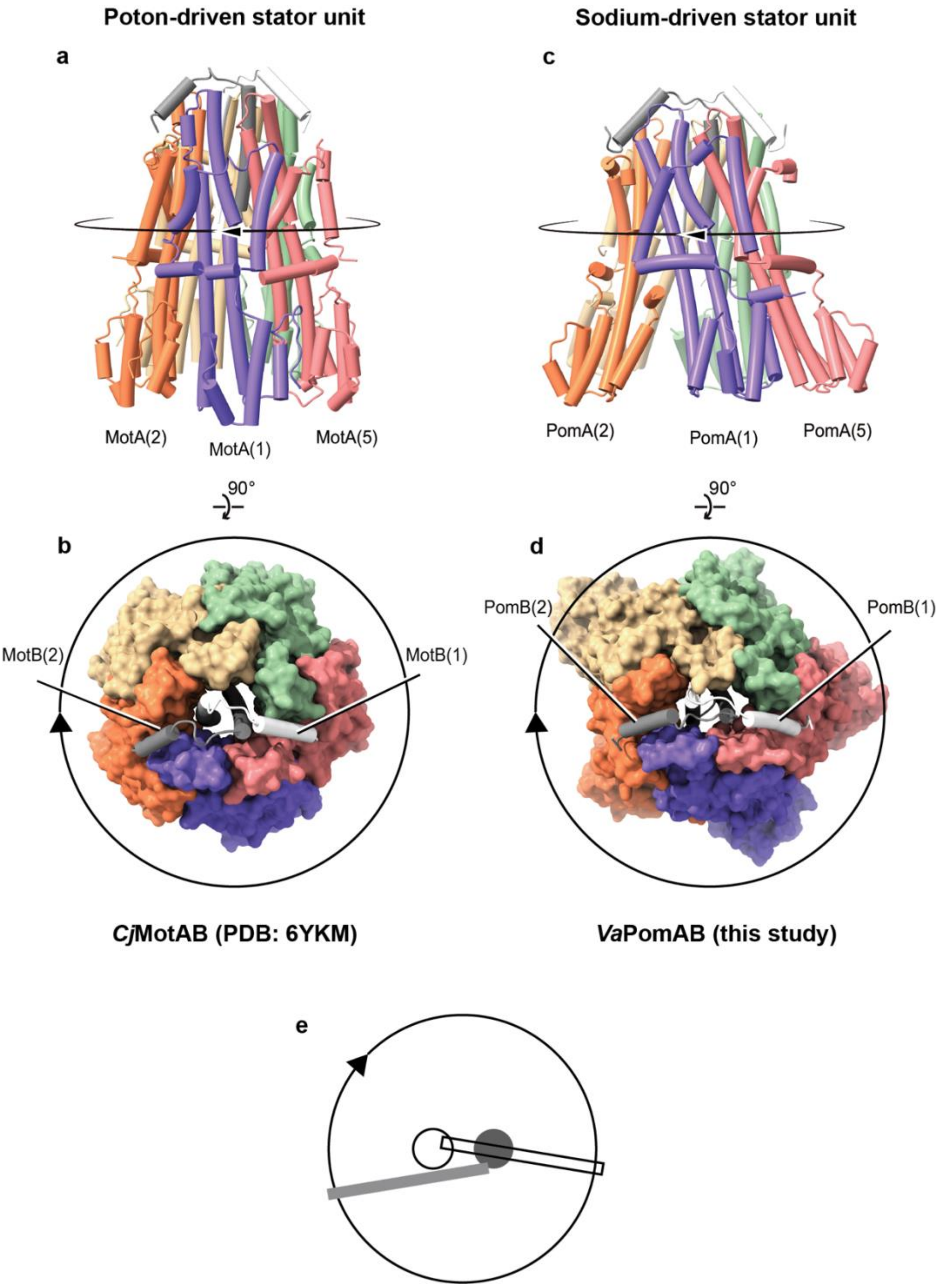
Poton-driven stator unit
Sodium-driven stator unit
a
c
MotA(2)
MotA(1)
MotA(5)
PomA(2)
PomA(1)
PomA(5)
90°
90°
b
d
MotB(2)
MotB(1)
PomB(2)
PomB(1)
*Cj*MotAB (PDB: 6YKM)
*Va*PomAB (this study)
e

Supplementary Fig. 13. H^+- and Na^+-driven stator units PomB/MotB plug motifs organization.

a, Side view of the proton-driven stator unit *Cj*MotAB in its auto-inhibited state.**b**, *Cj*MotAB viewed from the top of the membrane. **c**, Side view of the sodium-driven stator *Va*PomAB in its auto-inhibited state. **d**, *Va*PomAB viewed from the top of the membrane. Rotational direction of the stator unit is indicated. **e**, The unique trans mode organization of the plug motifs tightly blocks the CW rotation of the stator unit.

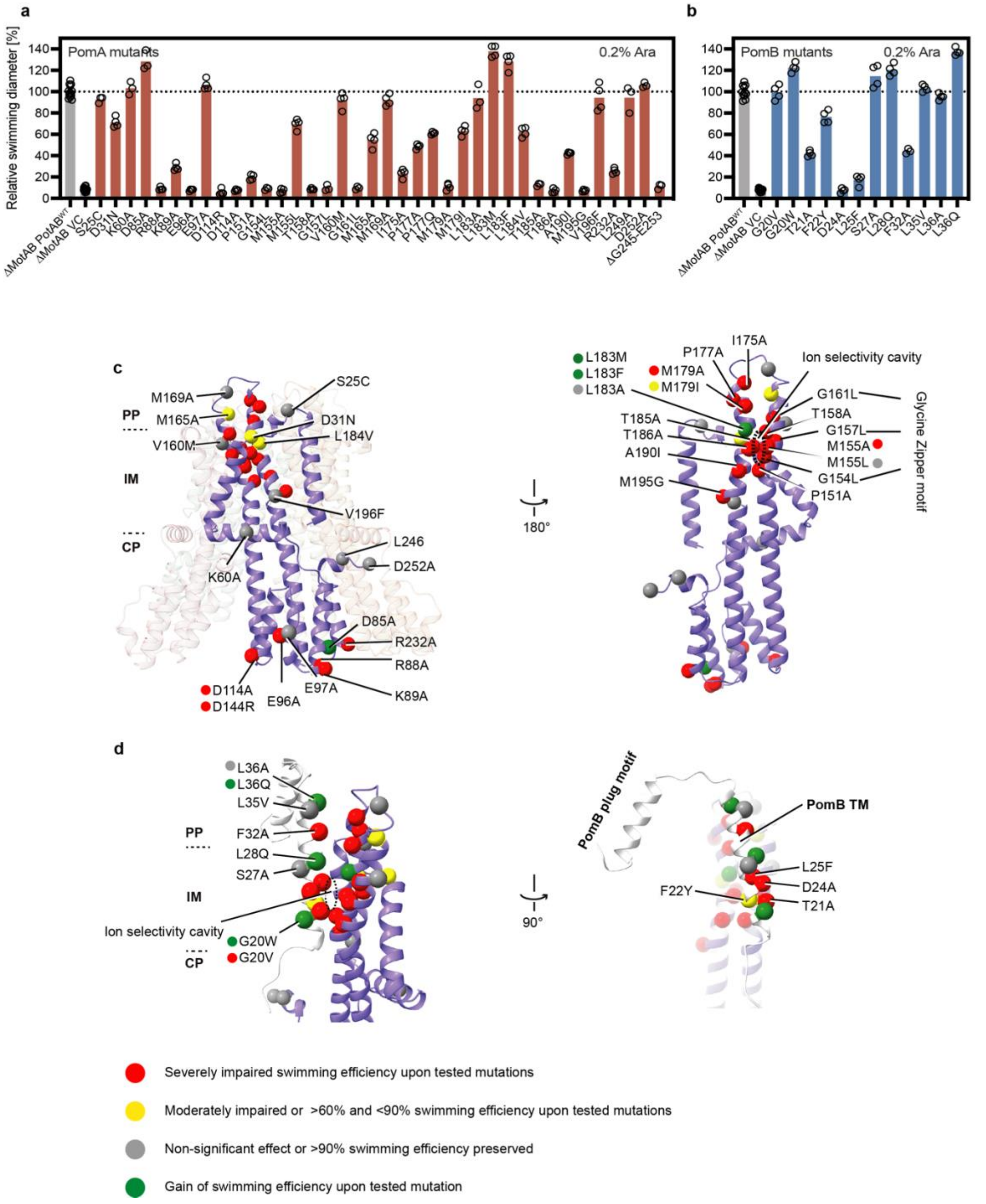

Supplementary Fig. 14. Mutational analysis for *Va*PomA and *Va*PomB plotted onto the *Va*PomAB structure.

a-**b**, The motility phenotypes of *Va*PotAB PomA (**a**) and PotB (**b**) point mutants were analyzed using soft-agar motility plates containing 0.2% agar. Source data are provided as a Source Data file. **c**-**d**, Swimming efficiency of the *Va*PotAB point mutants, showing the mutated residues as Cα spheres on the PomA (purple) and PomB (white) structure.

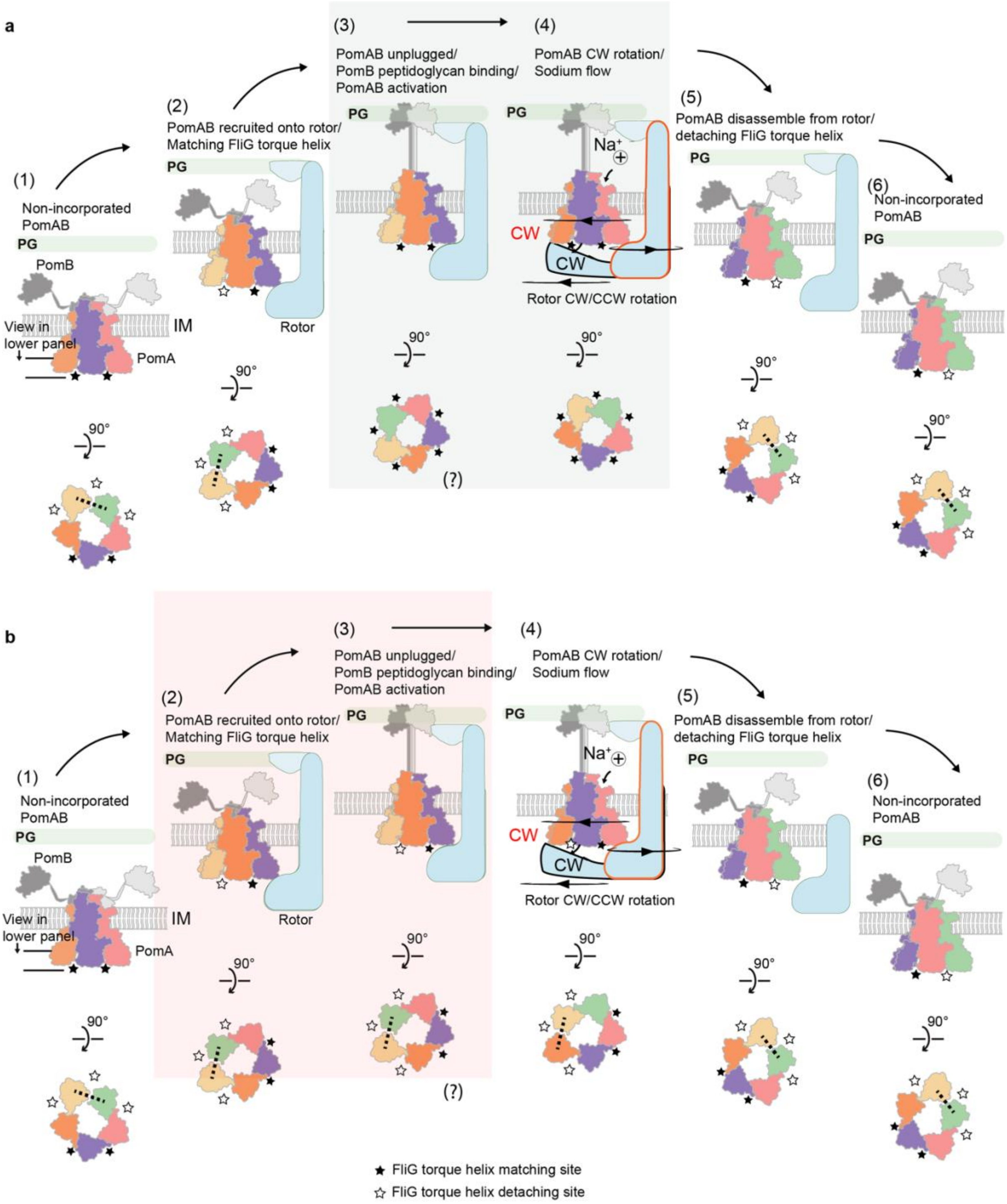
a
(1)
Non-incorporated PomAB
PG
PomB
View in lower panel
IM
PomA
(2)
PomAB recruited onto rotor/ Matching FliG torque helix
Rotor
(3)
PomAB unplugged/ PomB peptidoglycan binding/ PomAB activation
(?)
(4)
PomAB CW rotation/ Sodium flow
Na+
CW
Rotor CW/CCW rotation
(5)
PomAB disassemble from rotor/ detaching FliG torque helix
(6)
Non-incorporated PomAB
90°
b
★ FliG torque helix matching site
☆ FliG torque helix detaching site

Supplementary Fig. 15. Conformational changes of PomA cytoplasmic domain during stator unit activation and disassembly from the rotor.

a, PomA cytoplasmic domain is asymmetric, and one site of the CH-CI detachment is indicated in dashed line. Inactive stator unit orients its cytoplasmic domain towards the rotor to contact FliG torque helix through FliG torque helix 'matching sites' ((1)-(2)). During the activation, all five CH-CI interactions established, and PomA cytoplasmic domain becomes symmetric ((3)-(4)). The rotor could rotate either CW or CCW direction, depending on how it interacts with the stator unit. Stator unit disassembly from the rotor when external torque is decreased ((5)-(6)). **b**, In this model, during the stator unit activation, PomA cytoplasmic domain remains asymmetric ((3)-(4)); one site of the CI helix attaches to the PI helix and the adjacent CI helix detaches from the PI helix, sequentially creating a FliG torque helix 'catching' site that interacts with the FliG torque helix.

a

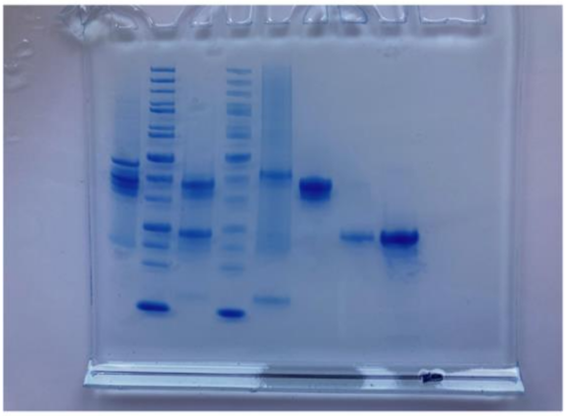

b

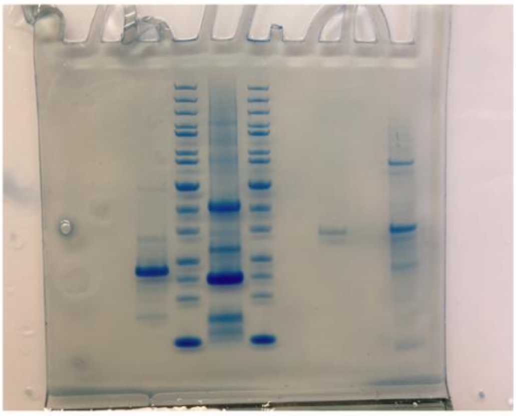

c

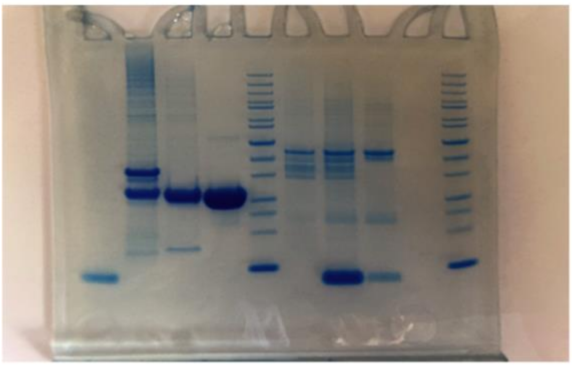

Supplementary Fig. 16. Uncropped and unprocessed SDS gels.

a, Uncropped and unprocessed SDS gels in supplementary Fig. 2b. **b**, Uncropped and unprocessed SDS gels in supplementary Fig. 3a. **c**, Uncropped and unprocessed SDS gels in supplementary Fig. 4a.

Supplementary Table 1. Cryo-EM data collection, refinement and validation statistics.

	***Va*PomAB**	***Va*PomAB (MSP1D1 nanodisc)**	***Va*PomAB (Saposin nanodisc)**
**Data collection and processing**			
Microscope	Titan Krios G2		
Voltage (kV)	300		
Magnification (nominal)	96,000x		
Total exposure (e^-/Å^2)	37.98	40.00	37.98
Exposure fractions (no.)	40		
Pixel size (Å)	0.832		
Movies used (no.)	6,444	3,798	1,823
Total picked particles (no.)	3,659,926	959,766	498,542
Final particles (no.)	923,963	66,296	38,438
Box size (pixels)	500	400	400
Symmetry imposed	C1		
Map resolution (Å) (FSC 0.143)	2.48	3.90	6.30
**Refinement**			
Model composition			
Non-hydrogen atoms	9,926	99,16	
Protein residues	1,312	1,312	
Solvent molecules	12	-	
B-factors (mean; Å^2)			
Protein	75.39	123.52	
Solvent	30.06	-	
R.m.s. deviations			
Bond lengths (Å)	0.006	0.006	
Bond angles (□)	0.874	1.288	
CC (mask)	0.83	0.79	
Refinement resolution (FSC map vs. model (masked)=0.143) (Å)	2.4	3.8	
**Validation**			
MolProbity score	2.04	2.08	
Poor rotamers (%)	0.00	0.09	
Ramachandran plot			
Favored (%)	97.61	97.23	
Allowed (%)	2.39	2.77	
Disallowed (%)	0.00	0.00	

Supplementary Table 2: bacterial strains

Number	Genotype	Source
n/a	NEB Dh5alpha (cloning strain)	NEB, Ipswich, MA, USA
TH437	Salmonella enterica serovar Typhimurium LT2	J. Roth (University of California, Davis)
EM10372	DmotAB (leaving first and last 15 bp)	Mònica Santiveri et al. 2020
EM12719	LT2 / hhd-pbad33-pomapotb59-e-coli (p46) (CmR)	This study
EM12720	LT2 / hhd-p47-pbad33-pomapotb59-samonella (p47) (CmR)	This study
EM12721	DmotAB / hhd-pbad33-pomapotb59-e-coli (p46) (CmR)	This study
EM12722	DmotAB / hhd-p47-pbad33-pomapotb59-samonella (p47) (CmR)	This study
EM12734	LT2 / pBAD33.1 (Adgene #36267; CmR)	This study
EM12735	DmotAB / pBAD33.1 (Adgene #36267; CmR)	This study
EM13301	DmotAB / pEM13101 (pBAD33.1-pomA(S25C)pomB, Samonella PG-binding domain, CmR)	This study
EM13302	DmotAB / pEM13102 (pBAD33.1-pomA(D31N)pomB, Samonella PG-binding domain, CmR)	This study
EM13303	DmotAB / pEM13103 (pBAD33.1-pomA(R88A)pomB, Samonella PG-binding domain, CmR)	This study
EM13304	DmotAB / pEM13104 (pBAD33.1-pomA(K89A)pomB, Samonella PG-binding domain, CmR)	This study
EM13305	DmotAB / pEM13105 (pBAD33.1-pomA(E96A)pomB, Samonella PG-binding domain, CmR)	This study
EM13306	DmotAB / pEM13106 (pBAD33.1-pomA(E97A)pomB, Samonella PG-binding domain, CmR)	This study
EM13307	DmotAB / pEM13107 (pBAD33.1-pomA(D114R)pomB, Samonella PG-binding domain, CmR)	This study
EM13308	DmotAB / pEM13108 (pBAD33.1-pomA(D114A)pomB, Samonella PG-binding domain CmR)	This study
EM13309	DmotAB / pEM13109 (pBAD33.1-pomA(P151A)pomB, Samonella PG-binding domain, CmR)	This study
EM13310	DmotAB / pEM13110 (pBAD33.1-pomA(M155A)pomB, Samonella PG-binding domain, CmR)	This study
EM13311	DmotAB / pEM13111 (pBAD33.1-pomA(M155L)pomB, Samonella PG-binding domain, CmR)	This study
EM13312	DmotAB / pEM13112 (pBAD33.1-pomA(T158A)pomB, Samonella PG-binding domain, CmR)	This study
EM13313	DmotAB / pEM13113 (pBAD33.1-pomA(V160M)pomB, Samonella PG-binding domain, CmR)	This study
EM13314	DmotAB / pEM13114 (pBAD33.1-pomA(M165A)pomB, Samonella PG-binding domain, CmR)	This study
EM13315	DmotAB / pEM13115 (pBAD33.1-pomA(M169A)pomB, Samonella PG-binding domain, CmR)	This study
EM13316	DmotAB / pEM13116 (pBAD33.1-pomA(I175A)pomB, Samonella PG-binding domain, CmR)	This study
EM13317	DmotAB / pEM13117 (pBAD33.1-pomA(P177A)pomB, Samonella PG-binding domain, CmR)	This study
EM13318	DmotAB / pEM13118 (pBAD33.1-pomA(P177Q)pomB, Samonella PG-binding domain, CmR)	This study
EM13319	DmotAB / pEM13119 (pBAD33.1-pomA(M179A)pomB, Samonella PG-binding domain, CmR)	This study
EM13320	DmotAB / pEM13120 (pBAD33.1-pomA(M179I)pomB, Samonella PG-binding domain, CmR)	This study
EM13321	DmotAB / pEM13121 (pBAD33.1-pomA(L183A)pomB, Samonella PG-binding domain, CmR)	This study

EM13322	DmotAB / pEM13122 (pBAD33.1-pomA(L183M)pomB, Samonella PG-binding domain, CmR)	This study
EM13323	DmotAB / pEM13123 (pBAD33.1-pomA(L183F)pomB, Samonella PG-binding domain, CmR)	This study
EM13324	DmotAB / pEM13124 (pBAD33.1-pomA(L184V)pomB, Samonella PG-binding domain, CmR)	This study
EM13325	DmotAB / pEM13125 (pBAD33.1-pomA(T186A)pomB, Samonella PG-binding domain, CmR)	This study
EM13326	DmotAB / pEM13126 (pBAD33.1-pomA(A190I)pomB, Samonella PG-binding domain, CmR)	This study
EM13327	DmotAB / pEM13127 (pBAD33.1-pomA(M195G)pomB, Samonella PG-binding domain, CmR)	This study
EM13328	DmotAB / pEM13128 (pBAD33.1-pomA(V196F)pomB, Samonella PG-binding domain, CmR)	This study
EM13329	DmotAB / pEM13129 (pBAD33.1-pomA(R232A)pomB, Samonella PG-binding domain, CmR)	This study
EM13330	DmotAB / pEM13130 (pBAD33.1-pomApomB(G20V), Samonella PG-binding domain, CmR)	This study
EM13331	DmotAB / pEM13131 (pBAD33.1-pomApomB(G20W), Samonella PG-binding domain, CmR)	This study
EM13332	DmotAB / pEM13132 (pBAD33.1-pomApomB(T21A), Samonella PG-binding domain, CmR)	This study
EM13333	DmotAB / pEM13133 (pBAD33.1-pomApomB(F22Y), Samonella PG-binding domain, CmR)	This study
EM13334	DmotAB / pEM13134 (pBAD33.1-pomApomB(D24A), Samonella PG-binding domain, CmR)	This study
EM13335	DmotAB / pEM13135 (pBAD33.1-pomApomB(L25F), Samonella PG-binding domain, CmR)	This study
EM13336	DmotAB / pEM13136 (pBAD33.1-pomApomB(S27A), Samonella PG-binding domain, CmR)	This study
EM13337	DmotAB / pEM13137 (pBAD33.1-pomApomB(L28Q), Samonella PG-binding domain, CmR)	This study
EM13338	DmotAB / pEM13138 (pBAD33.1-pomApomB(F32A), Samonella PG-binding domain, CmR)	This study
EM13339	DmotAB / pEM13139 (pBAD33.1-pomApomB(L35V), Samonella PG-binding domain, CmR)	This study
EM13340	DmotAB / pEM13140 (pBAD33.1-pomApomB(L36A), Samonella PG-binding domain, CmR)	This study
EM13341	DmotAB / pEM13141 (pBAD33.1-pomApomB(L36Q), Samonella PG-binding domain, CmR)	This study
EM13783	DmotAB / pEM13778 (pBAD33.1-pomA(K60A)pomB, Samonella PG-binding domain CmR)	This study
EM13784	DmotAB / pEM13779 (pBAD33.1-pomA(T185A)pomB, Samonella PG-binding domain CmR)	This study
EM13785	DmotAB / pEM13780 (pBAD33.1-pomA(L249A)pomB, Samonella PG-binding domain CmR)	This study
EM13786	DmotAB / pEM13781 (pBAD33.1-pomA(D252A)pomB, Samonella PG-binding domain CmR)	This study
EM13787	DmotAB / pEM13782 (pBAD33.1-pomA(ΔAA245-253)pomB, Samonella PG-binding domain CmR)	This study
EM14991	DmotAB / pEM14969 (pBAD33.1-pomA(D85A)pomB, Samonella PG-binding domain, CmR)	This study
EM14992	DmotAB / pEM14970 (pBAD33.1-pomA(G154L)pomB, Samonella PG-binding domain, CmR)	This study
EM14993	DmotAB / pEM14971 (pBAD33.1-pomA(G157L)pomB, Samonella PG-binding domain, CmR)	This study
EM14994	DmotAB / pEM14972 (pBAD33.1-pomA(G161L)pomB, Samonella PG-binding domain, CmR)	This study

Supplementary Table 3: Primers

Number	Name	Sequence 5'-3'
5593	Va-PomAS25C-fwd	GCTTGGTGGCtGCATCGGCATGTTTGTC
5594	Va-PomAS25C-rv	GACAAACATGCCGATGCaGCCACCAAGC
5595	Va-PomAD31N-fwd	CATGTTTGTCaATGTCACGTCGATCC
5596	Va-PomAD31N-rv	GGATCGACGTGACATtGACAAACATG
5597	Va-PomAR88A-fwd	GATGCGGCGgccAAAGGTGGTTTTCTTG
5598	Va-PomAR88A-rv	CAAGAAAACCACCTTTggcCGCCGCATC
5599	Va-PomAK89A-fwd	GATGCGGCGCGTgcgGGTGGTTTTC
5600	Va-PomAK89A-rv	GAAAACCACCcgcACGCGCCGCATC
5601	Va-PomAE96A-fwd	CTTGCTCTTGcgGAGATGGAAATAAAC
5602	Va-PomAE96A-rv	GTTTATTTCCATCTCcgCAAGAGCAAG
5603	Va-PomAE97A-fwd	CTTGCTCTTGAAGccATGGAAATAAAC
5604	Va-PomAE97A-rv	GTTTATTTCCATggCTTCAAGAGCAAG
5605	Va-PomAD114R-fwd	GATCTACTGGTTcgcGGCCATGATGC
5606	Va-PomAD114R-rv	GCATCATGGCCgcgAACCAGTAGATC
5607	Va-PomAD114A-fwd	GATCTACTGGTTGcgGGCCATGATG
5608	Va-PomAD114A-rv	CATCATGGCCcgCAACCAGTAGATC
5609	Va-PomAP151A-fwd	GGCGACGTTGCTgCcGCGATGGGAATG
5610	Va-PomAP151A-rv	CATTCCCATCGCgGcAGCAACGTCGCC
5611	Va-PomAM155A-fwd	CTGCGATGGGAgcGATTGGCACCTTG
5612	Va-PomAM155A-rv	CAAGGTGCCAATCgcTCCCATCGCAG
5613	Va-PomAM155L-fwd	CTGCGATGGGAcTGATTGGCACCTTG
5614	Va-PomAM155L-rv	CAAGGTGCCAATCAgTCCCATCGCAG
5615	Va-PomAT158A-fwd	GGGAATGATTGGCgCgTTGGTTGGTC
5616	Va-PomAT158A-rv	GACCAACCAAcGcGCCAATCATTCCC
5617	Va-PomAV160M-fwd	GATTGGCACCTTGaTgGGTCTTGTTG
5618	Va-PomAV160M-rv	CAACAAGACCcAtCAAGGTGCCAATC
5619	Va-PomAM165A-fwd	GGTCTTGTTGCGgcGCTTTCAAACATG
5620	Va-PomAM165A-rv	CATGTTTGAAAGCgcCGCAACAAGACC
5621	Va-PomAM169A-fwd	GCTTTCAAACgcGGATGACCCTAAAGC
5622	Va-PomAM169A-rv	GCTTTAGGGTCATCCgcGTTTGAAAGC
5623	Va-PomAI175A-fwd	GGATGACCCTAAAGCGgcgGGACCAGC
5624	Va-PomAI175A-rv	GCTGGTCCcgcCGCTTTAGGGTCATCC
5625	Va-PomAP177A-fwd	CTAAAGCGATTGGAgCAGCAATGGCCG
5626	Va-PomAP177A-rv	CGGCCATTGCTGcTCCAATCGCTTTAG
5627	Va-PomAP177Q-fwd	CTAAAGCGATTGGACaAGCAATGGCCG
5628	Va-PomAP177Q-rv	CGGCCATTGCTtGTCCAATCGCTTTAG
5629	Va-PomAM179A-fwd	GATTGGACCAGCAgcGGCCGTTGCAC
5630	Va-PomAM179A-rv	GTGCAACGGCCgcTGCTGGTCCAATC
5631	Va-PomAM179I-fwd	GATTGGACCAGCAATtGCCGTTGCAC
5632	Va-PomAM179I-rv	GTGCAACGGCaATTGCTGGTCCAATC
5633	Va-PomAL183A-fwd	GGCCGTTGCAgcCTTGACCACATTG
5634	Va-PomAL183A-rv	CAATGTGGTCAAGgcTGCAACGGCC
5635	Va-PomAL183M-fwd	GGCCGTTGCAaTgTTGACCACATTG
5636	Va-PomAL183M-rv	CAATGTGGTCAAcAtTGCAACGGCC

5637	Va-PomAL183F-fwd	GGCCGTTGCAtTtTTGACCACATTGTATG
5638	Va-PomAL183F-rv	CATACAATGTGGTCAAaAaTGCAACGGCC
5639	Va-PomAL184V-fwd	GGCCGTTGCACTCgTGACCACATTG
5640	Va-PomAL184V-rv	CAATGTGGTCAcGAGTGCAACGGCC
5641	Va-PomAT186A-fwd	GCACTCTTGACCgCATTGTATGGCG
5642	Va-PomAT186A-rv	CGCCATACAATGcGGTCAAGAGTGC
5643	Va-PomAA190I-fwd	CACATTGTATGGCattATCCTGTCC
5644	Va-PomAA190I-rv	GGACAGGATaatGCCATACAATGTG
5645	Va-PomAM195G-fwd	GATCCTGTCCAATggGGTGTTTTTCCC
5646	Va-PomAM195G-rv	GGGAAAAACACCccATTGGACAGGATC
5647	Va-PomAV196F-fwd	CCAATATGtTcTTTTTCCCTATTGCGG
5648	Va-PomAV196F-rv	CCGCAATAGGGAAAAAgAaCATATTGG
5649	Va-PomAR232A-fwd	GCCAAAACCCGgcAGTGATCGATAG
5650	Va-PomAR232A-rv	CTATCGATCACTgcCGGGTTTTGGC
5651	Va-PotBG20V-fwd	CCGTTATGGATGGtGACATTCGCAG
5652	Va-PotBG20V-rv	CTGCGAATGTCaCCATCCATAACGG
5653	Va-PotBG20W-fwd	CCGTTATGGATGtGGACATTCGCAG
5654	Va-PotBG20W-rv	CTGCGAATGTCCaCATCCATAACGG
5655	Va-PotBT21A-fwd	GGATGGGGgCATTCGCAGATTTGATG
5656	Va-PotBT21A-rv	CATCAAATCTGCGAATGcCCCCATCC
5657	Va-PotBF22Y-fwd	GATGGGGACATaCGCAGATTTGATG
5658	Va-PotBF22Y-rv	CATCAAATCTGCGtATGTCCCCATC
5659	Va-PotBD24A-fwd	GATGGGGACATTCGCAGcTTTGATGTC
5660	Va-PotBD24A-rv	GACATCAAAgCTGCGAATGTCCCCATC
5661	Va-PotBL25F-fwd	GGACATTCGCAGATTTtATGTCGCTGC
5662	Va-PotBL25F-rv	GCAGCGACATaAAATCTGCGAATGTCC
5663	Va-PotBS27A-fwd	CGCAGATTTGATGgCGCTGCTGATGTG
5664	Va-PotBS27A-rv	CACATCAGCAGCGcCATCAAATCTGCG
5665	Va-PotBL28Q-fwd	GATTTGATGTCGCaGCTGATGTGTTTC
5666	Va-PotBL28Q-rv	GAAACACATCAGCtGCGACATCAAATC
5667	Va-PotBF32A-fwd	CTGCTGATGTGTgcCTTTGTTCTTCTG
5668	Va-PotBF32A-rv	CAGAAGAACAAAGgcACACATCAGCAG
5669	Va-PotBL35V-fwd	GTGTTTCTTTGTTgTTCTGCTCTCG
5670	Va-PotBL35V-rv	CGAGAGCAGAAcAACAAAGAAACAC
5671	Va-PotBL36A-fwd	CTTTGTTCTTgcGCTCTCGTTTTCTG
5672	Va-PotBL36A-rv	CAGAAAACGAGAGCgcAAGAACAAAG
5673	Va-PotBL36Q-fwd	CTTTGTTCTTCaGCTCTCGTTTTCTG
5674	Va-PotBL36Q-rv	CAGAAAACGAGAGCtGAAGAACAAAG
5756	Va-PomAS25C-fwd_rth	tGCATCGGCATGTTTGTCGATG
5757	Va-PomAS25C-rv_rth	GCCACCAAGCACCATCGCC
5758	Va-PomAR88A-fwd:rth	gccAAAGGTGGTTTTCTTGCTC
5759	Va-PomAR88A-rv_rth	CGCCGCATCGGCCATTTCC
5760	Va-PomAK89A-fwd_rth	gcgGGTGGTTTTCTTGCTCTTG
5761	Va-PomAK89A-rv_rth	ACGCGCCGCATCGGCCATTTC
5762	Va-PomAE96A-fwd_rth	cgGAGATGGAAATAAACAACAC
5763	Va-PomAE96A-rv_rth	CAAGAGCAAGAAAACCACC
5764	Va-PomAM155A-fwd	gcGATTGGCACCTTGGTTGGTC
5765	Va-PomAM155A-rv	TCCCATCGCAGGAGCAACG

5766	Va-PotBF22Y-fwd_rth	aCGCAGATTTGATGTCGCTGC
5767	Va-PotBF22Y-rv_rth	ATGTCCCCATCCATAACGG
6299	Va-PomA_K60A-fwd	GGTGCGACAgccATTGCTGGCAAAGCC
6300	Va-PomA_T185A -fwd	CGTTGCACTCTTGgCCACATTGTATGGC
6301	Va-PomA_L249A-fwd	CGTGCCgcTGAGATTGACGAGTAACTTG
6302	Va-PomA_D252A-fwd	CTTGAGATTGcCGAGTAACTTGGAGAG
6303	Va-PomA_DeltaC - fwd_rth	TAACTTGGAGAGTCGTGATG
6304	Va-PomA_K60A-rv	GGCTTTGCCAGCAATggcTGTCGCACC
6305	Va-PomA_T185A -rv	GCCATACAATGTGGcCAAGAGTGCAACG
6306	Va-PomA_L249A-rv	CAAGTTACTCGTCAATCTCAgcGGCACG
6307	Va-PomA_D252A-rv	CTCTCCAAGTTACTCGgCAATCTCAAG
6308	Va-PomA_DeltaC -rv_rth	TTCATTGAGGTAGTTCTTCAAG
6299	Va-PomA_K60A-fwd	GGTGCGACAgccATTGCTGGCAAAGCC
6300	Va-PomA_T185A -fwd	CGTTGCACTCTTGgCCACATTGTATGGC
6301	Va-PomA_L249A-fwd	CGTGCCgcTGAGATTGACGAGTAACTTG
6302	Va-PomA_D252A-fwd	CTTGAGATTGcCGAGTAACTTGGAGAG
6303	Va-PomA_DeltaC - fwd_rth	TAACTTGGAGAGTCGTGATG
6304	Va-PomA_K60A-rv	GGCTTTGCCAGCAATggcTGTCGCACC
6305	Va-PomA_T185A -rv	GCCATACAATGTGGcCAAGAGTGCAACG
6306	Va-PomA_L249A-rv	CAAGTTACTCGTCAATCTCAgcGGCACG
6307	Va-PomA_D252A-rv	CTCTCCAAGTTACTCGgCAATCTCAAG
6308	Va-PomA_DeltaC -rv_rth	TTCATTGAGGTAGTTCTTCAAG
6540	p43_fwd	ATGATTGGCACCTTGGTTGG
6541	p43_rev	ATAATGACGAATGCAAAACC
6542	gBlock_p43_fwd	ggttttgcattcgtcattatGGCGATGGTGCTTGGTGG
6543	gBlock_p43_rev	ccaaccaaggtgccaatcatTCCCATCGCAGGAGCAAC
6544	p47_fwd	TCGCAGATTTGATGTCGCTG
6545	p47_rev	GCCATACAATGTGGTCAAGA
6546	gBlock_p47_fwd	tcttgaccacattgtatggcGCGATCCTGTCCAATATG
6547	gBlock_p47_rev	cagcgacatcaaatctgcgaATGTCCCCATCCATAACG
7107	Va-PomA_D85A-fwd-rth	TGCGGCGCGTAAAGGTGGTT
7108	Va-PomA_D85A-rv-rth	gCGGCCATTTCCACAATTTTTGC
7139	3'-GA-pomA-D85-20_rev	CCCTGCTGTTGGGTGTAATC
7140	3'-GA-D85-60_rev	TTTTTTCAGCTCGTCGATATTCGGCTGCTTTTCCACTTCACCCTGCTGTTGGGTGTA ATC
7141	5'-GA-G161-G161_fwd	ACGTTGCTCCTGCGATGGGAATGATTGGCACCTTGGTTctTCTTGTTGCGATGCTTTC AA
7142	3'-GA-G161-20lin1_rev	AACCAAGGTGCCAATCATTC
7143	5'-GA-G157-60_fwd	GCGCCTTTGGCGACGTTGCTCCTGCGATGGGAATGATTctCACCTTGGTTGGTCTTGT TG
7144	5'-GA-G154-60_fwd	TGTATTTCGCGCCTTTGGCGACGTTGCTCCTGCGATGctAATGATTGGCACCTTGGTT GG
7145	3'-GA-G154-20lin1_rev	CATCGCAGGAGCAACGTCGC
Sequencing primers		
3117	5'_pBAD24_seq_fw	cgggaccaaagccatgacaa
440	3'-pTrc-seq-rv	ggcaaattctgttttatcagac
5497	pomA_Va_check-fwd	GCCTTCATGTTTAAAGCGGA
5498	pomB_Va_check-fwd	GTGTTTCTTTGTTCTTCTGC

gBlocks		
128	p43_gBlock	GGCGATGGTGCTTGGTGGCAGCATCGGCATGTTTGTCGATGTCACGTCGATCCTTATTGTCGTTGGTGGCTCAATATTCGTCGTGTTGATGAAGTTCACAATGGGACAGTTTTTTGGTGCGACAgccATTGCTGGCAAAGCCTTCATGTTTAAAGCGGATGAACCCGAAGACCTGATCGCAAAAATTGTGGAAATGGCCGATGCGGCGCGTAAAGGTGGTTTTCTTGCTCTTGAAGAGATGGAAATAAACAACACATTCATGCAGAAAGGCATTGATCTACTGGTTGATGGCCATGATGCCGACGTTGTGAGAGCGGCACTCAAAAAAGACATCGCGCTTACGGATGAACGACATACGCAAGGTACTGGTGTATTTCGCGCCTTTGGCGACGTTGCTCCTGCGATGGGA
129	p47_gBlock	GCGATCCTGTCCAATATGGTGTTTTTCCCTATTGCGGATAAACTTTCTCTTCGCCGTGACCAAGAAACGCTAAATCGCCGTTTGATCATGGATGGCGTATTAGCGATTCAAGATGGCCAAAACCCGCGAGTGATCGATAGTTACTTGAAGAACTACCTCAATGAATAACTTGGAGAGTCGTGATGGATGATGAAGATAACAAATGCGATTGTCCGCCACCTGGCCTCCCGTTATGGATGGGGACAT
