# Supplementary material for: Ion selectivity and rotor coupling of the Vibrio flagellar sodium-driven stator unit
Source: Nat Commun. 2023 Jul 27;14:4411. doi: 10.1038/s41467-023-39899-z (PMC10374538; doi:10.1038/s41467-023-39899-z)
Supplement: Supplementary file 3 — Description of Additional Supplementary Files [file 41467_2023_39899_MOESM3_ESM.pdf]

Description of Additional Supplementary Files

Supplementary Movie 1: MD simulations of Na+ translocation pathway and dynamics of PomB D24. See also Supplementary Figure 6.

Supplementary Movie 2: Hydration of T33 and the Na+ translocation pathway obtained from explicit solvent MD simulations. See also Supplementary Figure 7.
