# Supplementary material for: Ion selectivity and rotor coupling of the Vibrio flagellar sodium-driven stator unit
Source: Nat Commun. 2023 Jul 27;14:4411. doi: 10.1038/s41467-023-39899-z (PMC10374538; doi:10.1038/s41467-023-39899-z)
Supplement: Supplementary file 6 — Reporting Summary [file 41467_2023_39899_MOESM6_ESM.pdf]

nature portfolio

Corresponding author(s): Nicholas M I Taylor

Last updated by author(s): Jun 15, 2023

# Reporting Summary

Nature Portfolio wishes to improve the reproducibility of the work that we publish. This form provides structure for consistency and transparency in reporting. For further information on Nature Portfolio policies, see our Editorial Policies and the Editorial Policy Checklist.

## Statistics

For all statistical analyses, confirm that the following items are present in the figure legend, table legend, main text, or Methods section.

| n/a | Confirmed | |
|---|---|---|
| ☐ | ☒ | The exact sample size (*n*) for each experimental group/condition, given as a discrete number and unit of measurement |
| ☐ | ☒ | A statement on whether measurements were taken from distinct samples or whether the same sample was measured repeatedly |
| ☒ | ☐ | The statistical test(s) used AND whether they are one- or two-sided<br>*Only common tests should be described solely by name; describe more complex techniques in the Methods section.* |
| ☒ | ☐ | A description of all covariates tested |
| ☒ | ☐ | A description of any assumptions or corrections, such as tests of normality and adjustment for multiple comparisons |
| ☒ | ☐ | A full description of the statistical parameters including central tendency (e.g. means) or other basic estimates (e.g. regression coefficient) AND variation (e.g. standard deviation) or associated estimates of uncertainty (e.g. confidence intervals) |
| ☒ | ☐ | For null hypothesis testing, the test statistic (e.g. *F*, *t*, *r*) with confidence intervals, effect sizes, degrees of freedom and *P* value noted<br>*Give P values as exact values whenever suitable.* |
| ☒ | ☐ | For Bayesian analysis, information on the choice of priors and Markov chain Monte Carlo settings |
| ☒ | ☐ | For hierarchical and complex designs, identification of the appropriate level for tests and full reporting of outcomes |
| ☒ | ☐ | Estimates of effect sizes (e.g. Cohen's *d*, Pearson's *r*), indicating how they were calculated |

*Our web collection on statistics for biologists contains articles on many of the points above.*

## Software and code

Policy information about availability of computer code

| | |
|---|---|
| Data collection | For cryo-EM: EPU<br>Scanning of motility plates: python 3.5, pyinsane 1.4.0<br>For MD simulation: Gromacs 2021.5 |
| Data analysis | For cryo-EM: CryoSPARC version 3.3.2 and version 4,<br>For motility studies: NIH ImageJ 1.48v, GrowthRates 4.3, ImageJ 1.53t, GraphPad Prism 9.4.1.<br>Other: Coot 0.9-pre, Phenix 1.13, Chimera X 1.6.1, Pymol 2.5.4 Bioinformatics Toolkit server (MUSCLE alignment), Python 3<br>For MD simulation: Gromacs 2021.5 gmx tools, GROmaps |

For manuscripts utilizing custom algorithms or software that are central to the research but not yet described in published literature, software must be made available to editors and reviewers. We strongly encourage code deposition in a community repository (e.g. GitHub). See the Nature Portfolio guidelines for submitting code & software for further information.

## Data

Policy information about availability of data

All manuscripts must include a data availability statement. This statement should provide the following information, where applicable:

- Accession codes, unique identifiers, or web links for publicly available datasets
- A description of any restrictions on data availability
- For clinical datasets or third party data, please ensure that the statement adheres to our policy

Atomic coordinates for VaPomAB in LMNG detergent and VaPomAB in MSP1D1 nanodisc were deposited in the Protein Data Bank under accession codes PDB: 8BRD and 8BRI, respectively. The corresponding electrostatic potential maps were deposited in the Electron Microscopy Data Bank (EMDB) under accession codes EMDB: EMD-16212and EMD-16215, respectively. The electrostatic potential map for full length VaPomAB in Saposin nanodisc was deposited in the EMDB under accession code EMDB: EMD-16214.

## Human research participants

Policy information about studies involving human research participants and Sex and Gender in Research.

| | |
|---|---|
| Reporting on sex and gender | N/A |
| Population characteristics | N/A |
| Recruitment | N/A |
| Ethics oversight | N/A |

Note that full information on the approval of the study protocol must also be provided in the manuscript.

# Field-specific reporting

Please select the one below that is the best fit for your research. If you are not sure, read the appropriate sections before making your selection.

☒ Life sciences ☐ Behavioural & social sciences ☐ Ecological, evolutionary & environmental sciences

For a reference copy of the document with all sections, see nature.com/documents/nr-reporting-summary-flat.pdf

# Life sciences study design

All studies must disclose on these points even when the disclosure is negative.

| | |
|---|---|
| Sample size | For cryo-EM: No sample size was calculated. Sample size was based on experience to have a number of micrographs/particles that, if possible, would result in a cryo-EM reconstruction that would allow atomic model construction.<br>For motility assay: No a priori sample sizes were calculated. Sample sizes were chosen according to our experience in similar experimental setups e.g. Santiveri, M. et al. (2020) Cell 183: 244-257.e16.; Ward et. al. (2018) Mol Microbiol 107: 94-103.; Spöring, I. et al. (2018) PLoS Biol 16: e2006989.; Renault, T.T. et al. (2017) Elife 6: e23136.; Fabiani, F.D. et al. (2017) PLoS Biol 15: e2002267. The exact sample size (n) are provided in the figure legends. |
| Data exclusions | No data was excluded from the analysis. |
| Replication | For cryo-EM: PomAB complex was expressed and purified several times, always obtaining the same purification profile. All datasets obtained from cryo-EM data collection were analyzed using different versions of cryoSPARC and the results were always consistent and replicable.<br>For motility assay: All reported experiments have been repeated at least 3 times with independent samples. All experimental results shown were reproducible. No exclusion criteria were pre-established. |
| Randomization | No experimental groups were formed/compared. Data was collected randomly in each set of experiments. |
| Blinding | The researchers were not blinded to sample identity. |

# Reporting for specific materials, systems and methods

We require information from authors about some types of materials, experimental systems and methods used in many studies. Here, indicate whether each material, system or method listed is relevant to your study. If you are not sure if a list item applies to your research, read the appropriate section before selecting a response.

## Materials & experimental systems

| n/a | Involved in the study |
| --- | --- |
| ☒ | ☐ Antibodies |
| ☒ | ☐ Eukaryotic cell lines |
| ☒ | ☐ Palaeontology and archaeology |
| ☒ | ☐ Animals and other organisms |
| ☒ | ☐ Clinical data |
| ☒ | ☐ Dual use research of concern |

## Methods

| n/a | Involved in the study |
| --- | --- |
| ☒ | ☐ ChIP-seq |
| ☒ | ☐ Flow cytometry |
| ☒ | ☐ MRI-based neuroimaging |
